# Metabolomic characterization of unintentional weight loss among community‐dwelling older Black and White men and women

**DOI:** 10.1111/acel.14410

**Published:** 2024-11-15

**Authors:** Shanshan Yao, Megan M. Marron, Samaneh Farsijani, Iva Miljkovic, George C. Tseng, Ravi V. Shah, Venkatesh L. Murthy, Anne B. Newman

**Affiliations:** ^1^ University of Pittsburgh Pittsburgh Pennsylvania USA; ^2^ Vanderbilt University Medical Center Nashville Tennessee USA; ^3^ University of Michigan Ann Arbor Michigan USA

**Keywords:** appetite, metabolism, metabolomics, sarcopenia, weight loss

## Abstract

This study aims to understand the metabolic mechanisms of unintentional weight loss in older adults. We investigated plasma metabolite associations of subsequent weight change over 2 years in 1536 previously weight stable participants (mean age 74.6 years, 50% women, 35% Black) from the Health, Aging and Body Composition (Health ABC) Study. Multinomial logistic regressions were used to examine associations of the 442 metabolites with weight loss with/without an intention and weight gain >3% annually relative to weight stability. The metabolite associations of unintentional weight loss differed from those of intentional weight loss and weight gain. Lower levels of aromatic amino acids, phospholipids, long‐chain poly‐unsaturated triglycerides, and higher levels of amino acid derivatives, poly‐unsaturated fatty acids, and carbohydrates were associated with higher odds of unintentional weight loss after adjusting for age, sex, race, and BMI categories. Prevalent diseases attenuated four and lower mid‐thigh muscle mass and poorer appetite each attenuated 2 of 77 identified metabolite associations by >20%, respectively. Other factors (e.g., energy expenditure, diet, and medication) attenuated all associations by <20%. While 16 metabolite associations were attenuated by 20%–48% when adjusting for all these risk factors, 47 metabolite associations remained significant. Altered amino acid metabolism, impaired mitochondrial fatty acid oxidation, and inflammaging implicated by identified metabolites appear to precede unintentional weight loss in Health ABC older adults. Furthermore, these pathways seem to be associated with prevalent diseases especially diabetes, lower muscle mass, and poorer appetite.

## Graphical abstract

Metabolomic analysis of older adults reveals distinct signatures associated with incident unintentional versus intentional weight loss. Data suggests mitochondrial dysfunction and inflammation may precede such unintentional weight loss.

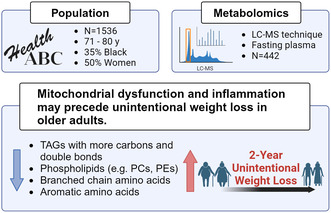

## 1 INTRODUCTION

Weight change is common among older adults aged 60 years and over worldwide, with the prevalence of weight changes >5% ranging from 35% to 66% over a period of 3–18 years (Guo et al., 2023; Haugsgjerd et al., 2017; Underland et al., 2022). Studies have reported that over 30% of older adults experience weight loss, which is more common than weight gain (Guo et al., 2023; Haugsgjerd et al., 2017; Lee et al., 2004; Underland et al., 2022). Weight change in older adults has been associated with a higher mortality risk, with weight loss posing a higher risk than weight gain (Park et al., 2018).

Weight loss in older adults can be either intentional or unintentional, each having different health implications. Intentional weight loss through lifestyle intervention can improve mobility and function in older adults (Albert et al., 2022; Santanasto et al., 2011). Older participants intended to lose weight generally reported better eating behaviors and more active lifestyles than did participants without an intention, independent of other health conditions (Lee et al., 2004). Conversely, unintentional weight loss often signals predisposing or unrecognized health problems, and is associated with a higher risk of adverse outcomes, including functional decline and higher mortality (de Stefani et al., 2018; Sahyoun et al., 2004; Wannamethee et al., 2005).

Understanding the pathways of weight change, particularly unhealthy weight change, among older adults is critical for predicting risks, guiding clinical interventions and developing therapeutic targets to promote healthy aging. Weight change in older adults has been associated with metabolic changes in aging, such as chronic inflammation, insulin resistance, hormone change, and mitochondrial dysfunction, which can lead to decreased muscle mass and subsequent weight loss (Palmer & Jensen, 2022). Given the complexity of body weight, influenced by genetic, environmental, and lifestyle factors, holistic approaches (e.g., metabolomics) are increasingly valuable in uncovering the metabolic mechanistic pathways involved.

Indeed, several metabolic pathways associated with intentional weight change in middle aged and older adults in clinical trials have been uncovered by metabolomics, including lipid metabolism (Almanza‐Aguilera et al., 2018; Bihlmeyer et al., 2021; Perez‐Cornago et al., 2014), amino acid turnover (Almanza‐Aguilera et al., 2018; Bihlmeyer et al., 2021; Perez‐Cornago et al., 2014; Quillen et al., 2020), inflammation (Porter Starr et al., 2019), and gut microbiota activity (Almanza‐Aguilera et al., 2018; Heianza et al., 2018; Lynch et al., 2023). However, the metabolic underpinnings of weight change in the general older, and especially multi‐ethnic population, are less explored. Considering the various risk factors and health outcomes associated with different types of weight change, the metabolic pathways may differ for intentional versus unintentional weight changes. Thus, using metabolomics data from the Health, Aging and Body Composition (Health ABC) study, we aim to identify metabolites associated with longitudinal change in body weight and to understand the potential underlying metabolic pathways of intentional weight loss, unintentional weight loss and weight gain in previously weight stable community‐dwelling older adults.

Moreover, previous efforts have identified some risk factors of unintentional weight loss, such as anorexia of aging or loss of appetite, physical activity and exercise, and dietary intake, which also have a metabolic basis (de Souto, 2022; Hubner et al., 2021). Lower lean mass was also observed for older adults who subsequently lost weight than those who did not (Newman et al., 2005). This suggests that there might be shared metabolic mechanisms of unintentional weight loss and these risk factors, which remains unexplored. Therefore, our secondary objective is to investigate whether potential risk factors including lifestyle and health conditions play a role in the metabolite associations of unintentional weight loss.

## 2 METHODS

### 2.1 Population

The Health ABC study is a prospective cohort (Visser et al., 2005). A total of 3075 Black and White older adults aged 70–79 years were recruited in March 1997 to July 1998 from Pittsburgh, Pennsylvania and Memphis, Tennessee. Participants were selected to be free of difficulty walking 1/4 mile, climbing up 10 steps, and with basic activities of daily living, with no reported use of a walking aid, no history of cancer treatment in the past 3 years, and no plans to move from the study area within 3 years after recruitment. The institutional review boards of the University of Pittsburgh and the University of Tennessee approved the study. All participants provided written informed consent. A total of 2469 participants who returned for the Year 2 clinic visit and had high‐quality venipuncture samples were selected for metabolomic profiling. Among the 2469 participants, we excluded 933 participants for the following reasons: 5 participants without fasting prior to blood draw; 134 with missing body weight at both Year 3 (1999–2000) and Year 4 (2000–2001) visits; 793 had gained or lost over 3% body weight from Year 1 to Year 2 (i.e., were not classified as weight stable prior to blood draw); and 1 with body weight change over 40% (10 times standard deviation from the mean). Thus, 1536 individuals were classified as weight stable prior to the Year 2 blood draw and were included in this study. Compared with other weight change groups, participants who had maintained a stable weight in the past year (from Year 1 to Year 2) were, in general, with similar appetite compared to the past year, fewer baseline smokers, better diet quality, higher energy expenditure, lower prevalence of diabetes and depression, and more likely to maintain weight stability during the follow‐up. (Table S1).

### 2.2 Body weight change

Body weight was measured at annual clinic visits from Year 2 (1998–1999) to Year 4 (2000–2001). Body height (cm) and weight (kg) were measured in light clothing without shoes on a calibrated balance beam scale. Weight change was calculated as the annual percentage change relative to Year 2 body weight. We aimed to detect participants with a short‐term weight change after the time of metabolomic profiling. Therefore, we classified participants based on their annual weight change during Years 2–4 as “weight gain” (>3% annual increase), “weight stable” (≤3% annual change), or “weight loss” (>3% annual decrease). This 3% cutoff was selected to exceed the coefficient of variation for dual‐energy X‐ray absorptiometry soft tissue mass (Economos et al., 1997; Figueroa‐Colon et al., 1998; Fuller et al., 1992) and has been used in previous studies (Deravi et al., 2022; Newman et al., 2005). The intention to lose weight was collected by a question: “At present, are you trying to lose weight?” at Year 2. Based on their intention to lose weight, we further classified participants into four weight change groups: weight stable (≤3% annual change), weight gain (“weight gain”), intentional weight loss (“weight loss” with self‐reported weight loss intention at Year 2), and unintentional weight loss (“weight loss” with self‐reported no weight loss intention at Year 2).

### 2.3 Metabolomics

Metabolites were measured in plasma collected at the Year 2 visit (1998–1999) after an overnight fast of ≥8 h using liquid chromatography‐mass spectrometry (LC–MS) methods at the Broad Institute (Cambridge, MA). Blood samples from the Health ABC participants had not been previously thawed and were stored at −80°C until metabolomic profiling. Four different LC–MS methodologies including (Haugsgjerd et al., 2017) polar metabolite profiling method that uses positive ion mode MS detection, (Guo et al., 2023) polar metabolite profiling method that uses negative ion mode MS detection, (Underland et al., 2022) lipid profiling method, and (Lee et al., 2004) intermediate polarity profiling method were employed to quantify metabolites. Metabolite values were LC–MS peak areas. Details of the metabolite profiling and quality control have been published elsewhere (Yao et al., 2024). To be noted, some metabolites were measured in more than one platform, which yielded 613 (520 unique) known and numerous unknown metabolites including duplicates. In this analysis we included 500 (442 unique) known metabolites, which were quantified in ≥90% of participants and had a coefficient of variation ≤10% (Yao et al., 2024). When duplicates of lipids or lipid‐like molecules correlated with weight change groups, we presented data from the lipid profiling (polar metabolite profiling with positive ion mode MS detection) method employing positive ion mode MS when it was available. For significant other metabolite duplicates, we reported findings from the platform demonstrating the lowest coefficient of variation. Missing values for metabolites included in the analyses were assumed to be due to true values being below detectable limits and were imputed as 50% of the lowest value detected for the respective metabolite (Yao et al., 2024). Metabolite values were log‐transformed and standardized for further analyses.

### 2.4 Participant characteristics

Age, sex, race, highest level of education, smoking behavior, and sleep duration were self‐reported and collected at baseline (i.e., Year 1). History or the presence of cardiovascular disease, hypertension, diabetes, and cancer were determined based on participants' self‐report of a physician diagnosis at baseline and Year 2. Participants were also categorized as having cardiovascular disease, hypertension, diabetes, cancer (any type except non‐melanoma skin cancer), and pulmonary disease if they were taking medication for these conditions. Other chronic diseases including peripheral artery disease, osteoarthritis, depression, and pulmonary disease were collected at baseline only. The number of prescription medications (anti‐hypertensive medications, lipid‐lowering medications, medications for diabetes, and anti‐inflammatory medications) used was calculated from a medication inventory at Year 2, which required participants to bring all prescription medications used in the last 2 weeks to the clinic visit. Obesity status was determined based on body mass index (BMI; kg/m^2^) calculated from measured body weight and height at Year 2, as follows: <25, 25–30, and ≥ 30 kg/m^2^. Total body fat was measured by dual‐energy X‐ray absorptiometry (Hologic QDR 4500A; Hologic, Bedford, MA) at Year 1 visit and percentage body fat mass was calculated by dividing total fat mass by total body mass. Mid‐thigh cross‐sectional muscle area was measured by Computed Tomography (9800 Advantage, General Electric Milwaukee, WI in Pittsburgh and Somatom Plus 4, Siemens, Erlangen, Germany, or PQ 2000S, Marconi Medical Systems, Cleveland, OH in Memphis) at Year 1 visit. Blood biomarkers of inflammation, interleukin‐6 (IL‐6) and C‐reactive protein (CRP), were measured using frozen stored serum collected after an overnight fast at Year 2 (Cesari et al., 2003). Current appetite was self‐reported appetite or desire to eat during the past month and was categorized into “very good”, “good”, and “moderate to poor or fluctuated”. Daily calories (Kcal/day), protein (g/day), fat (g/day), and carbohydrates (g/day) intake from food at Year 2 were determined using a 108‐item interviewer administered food frequency questionnaire estimating usual nutrient intake over the past year and was developed for the Health ABC study by Block Dietary Data Systems (Berkeley, CA) using food lists obtained from a 24 h recall among participants who were ages 65 or older, black or white race, and living in the Northeastern or Southern United States from the Third National Health and Nutrition Examination Survey (Marron et al., 2019). Daily fat (g/day) and protein intake (g/day) adjusted for total energy intake were calculated using the residual method (Willett et al., 1997). A Healthy Eating Index (HEI) score was also calculated (Lee et al., 2004). Physical activity was defined by a calculated energy expenditure (kcal/kg/week) in walking and climbing stairs at the Year 2 visit. To calculate energy expenditure in climbing stairs, we assigned 4.0 kcal/kg/h for stair climbing plus an additional 1.0 kcal/kg/h for carrying a load like laundry, groceries, or an infant. For energy expenditure in walking for exercise, we assigned 4.0 kcal/kg/h for walking briskly, 3.0 for walking at moderate pace, and 2.0 for strolling (Yao et al., 2024).

### 2.5 Statistical analysis

We summarized baseline characteristics of the study sample according to the weight change groups using mean (standard deviation, SD) or median (interquartile range) for continuous variables and frequency (proportion) for categorical variables. We tested the statistical difference in baseline characteristics by the one‐way analysis of variance or the Kruskal–Wallis rank sum test for continuous variables and the Pearson's Chi‐squared test for categorical variables.

We first conducted single metabolite analysis to identify metabolites that are associated with different weight change groups relative to weight stable in older adults using multinomial logistic analysis after adjusting for age, race, and sex. In this basal model, we only adjusted for age, race, and sex because we wanted to first identify all metabolites related to weight change patterns in the older adults, not just metabolites associated above and beyond certain risk factors. We then adjusted for Year 2 BMI categories in the model to identify metabolites associated with weight change independent of initial BMI category. We checked the non‐linear relationship with weight change groups by including a square term in the models for each metabolite. We also conducted single metabolite analyses stratified by race–sex groups, as well as by obesity status, and tested the interaction term of each of these factors with metabolites for differences in the metabolite associations with unintentional weight loss. After identifying metabolites associated with weight change, we then determined whether associations would be attenuated after further adjusting for known risk factors associated with weight change in older adults (Cao et al., 2019; Sahyoun et al., 2004). Potential confounders including baseline smoking, total body fat, percentage body fat, total mid‐thigh muscle area, appetite, dietary quality (HEI), daily calories, protein, and fat intake, pro‐inflammation biomarkers (IL‐6 and/or CRP), physical activity (energy expenditure), prevalent diseases (cardiovascular disease, hypertension, cancer, diabetes, and pulmonary disease), and number of prescription medications were selected a priori based on known risk factors of weight change and metabolism in older adults reported in literature (Cao et al., 2019; Lee et al., 2004; Sahyoun et al., 2004). Total body fat was not adjusted in the fully adjusted model due to its high correlation with BMI. We examined the extent to which these variables explained the BMI‐adjusted associations between metabolites and weight change using percent attenuation calculated as 100*(*β*1 − *β*2)/*β*1, where *β*1 is the age, race, sex, and BMI‐adjusted beta coefficient between weight change and metabolite values and *β*2 is the beta coefficient after further adjusting for other important risk factors. The significance level was assumed to be 0.05. To account for multiple comparisons, we used a Benjamini‐Hochberg correction with 5% false discovery rate (FDR) (Benjamini & Hochberg, 1995). All statistical analyses were conducted using R software version 4.2.1 (R Foundation for Statistical Computing).

## 3 RESULTS

Participants were, on average, 74.7 years old at Year 2 visit, including 50% men, and 35% reporting Black race. Weight change groups varied by race, education, smoking, body composition, IL‐6, CRP, and number of prescription medications. Older adults with unintentional weight loss were more likely to be Black, smokers, less overweight or obese, and had lower education, energy expenditure, total body fat, and percentage body fat. They also had smaller mid‐thigh muscle areas, higher calorie, and dietary fat intake, and higher IL‐6 levels compared to those with stable weight. Conversely, those with intentional weight loss were more likely to be overweight and obese, with higher total and percentage body fat, larger mid‐thigh muscle areas, higher CRP levels, and more prescription medications, but lower calorie, carbohydrate, and dietary fat intake. Weight gainers were less likely to be men compared to those with stable weight. (Table 1) Exploratory analysis showed no significant differences in clinical blood kidney, blood glucose, and lipid biomarkers across weight change groups. Participants with unintentional weight loss had lower baseline leptin levels than other groups. (Table S2).

**TABLE 1:** Characteristics of participants from the Health, Aging, and Body Composition study by weight change groups characteristics from Year 2 visit are presented except for otherwise indicated.

Characteristic	Weight change group				
	Overall	Intentional weight loss	Unintentional weight loss	Weight gain	Weight stable
*N*	1536	99	220	237	980
*Mean* (*SD*) *or Median* [*Q1*, *Q3*] *or n* (*%*)					
Age	74.7 (2.9)	75.0 (3.0)	74.9 (2.8)	74.6 (2.7)	74.6 (2.9)
Race, Black*	532 (35%)	38 (38%)	90 (41%)	91 (38%)	313 (32%)
Sex, Men	775 (50%)	49 (49%)	109 (50%)	111 (47%)	506 (52%)
More than high school education*	1202 (78%)	82 (84%)	155 (71%)	184 (78%)	781 (80%)
Baseline smoker*	130 (8%)	5 (5%)	31 (14%)	19 (8%)	75 (8%)
Baseline sleep hours/night	6.9 (1.3)	6.7 (1.6)	7.0 (1.5)	6.9 (1.4)	6.9 (1.3)
BMI category*					
<25 kg/m^2^	508 (33%)	8 (8%)	99 (45%)	80 (34%)	321 (33%)
25–30 kg/m^2^	653 (43%)	58 (59%)	82 (37%)	108 (46%)	405 (41%)
≥30 kg/m^2^	375 (24%)	33 (33%)	39 (18%)	49 (21%)	254 (26%)
Baseline total body fat(kg)*	26.7 (8.5)	30.2 (7.3)	24. 8 (7.8)	26.3 (8.1)	26.9 (8.7)
Baseline %body fat*	34.9 (7.6)	37.4 (6.8)	33.9 (7.6)	35.0 (7.7)	34.9 (7.7)
Baseline mid‐thigh muscle area (cm‐sq)*	224.7 (55.9)	233.5 (56.6)	214.8 (51.7)	223.1 (56.6)	226.5 (56.4)
Appetite*					
Very good	665 (44%)	40 (40%)	84 (39%)	99 (42%)	442 (46%)
Good	572 (38%)	45 (45%)	71 (33%)	90 (39%)	366 (38%)
Moderate to poor or fluctuating appetite	280 (18%)	14 (14%)	60 (28%)	44 (19%)	162 (17%)
Healthy Eating Index score	70.3 (11.9)	71.0 (11.5)	69.4 (12.3)	70.0 (12.2)	70.5 (11.7)
Calories (Kcal/day)	1744.2 [1355.6‐2249.9]	1650.1 (1342.3‐2206.9)	1839.7 (1463.6‐2426.4)	1729.4 (1292.5‐2309.2)	1743.5 (1350.8‐2208.0)
Daily macronutrients intake adjusted for energy intake					
Protein (g/day)	65.5 (58.8–75.0)	63.5 (56.4–73.5)	64.9 (56.9–72.2)	65.9 (58.5–76.9)	65.9 (59.6–74.9)
Fat (g/day)	70.8 (61.4–79.9)	74.1 (62.7–85.6)	40.8 (62.2–79.3)	70.6 (62.2–79.6)	70.6 (60.6–79.6)
Carbohydrates (g/day)	247.2 (226.5–270.7)	242.8 (212.9–265.2)	248.2 (228.0–271.5)	249.1 (227.4–270.4)	247.4 (226.6–271.1)
Energy expenditure from physical activity (kcal/kg/week)	3.3 (0.5–10.1)	3.5 (1.0–8.0)	2.7 (0.1–8.5)	2.5 (0.0–9.8)	3.5 (0.5–10.7)
Blood inflammation biomarkers					
Interleukin‐6 (pg/mL)	2.2 (1.5–3.7)	2.3 (1.6–4.2)	2.5 (1.6–4.1)	2.2 (1.4–4.0)	2.2 (1.4–3.5)
C‐reactive protein (μg/mL)*	3.0 (1.2–6.2)	3.7 (1.7–7.5)	2.7 (1.3–6.2)	3.4 (1.5–6.3)	2.8 (1.2–6.0)
Prevalent disease					
Year 2					
Cardiovascular disease	412 (27%)	19 (19%)	64 (29%)	60 (25%)	269 (27%)
Hypertension	794 (52%)	58 (59%)	118 (54%)	127 (54%)	491 (50%)
Diabetes	595 (39%)	39 (39%)	90 (41%)	99 (42%)	367 (37%)
Cancer	278 (18%)	18 (18%)	42 (19%)	41 (17%)	177 (18%)
Baseline					
Peripheral artery disease	67 (4%)	1 (1%)	11 (5%)	13 (6%)	42 (4%)
Osteoarthritis	156 (10%)	11 (11%)	30 (14%)	19 (8%)	96 (10%)
Depression	132 (9%)	8 (8%)	11 (5%)	25 (11%)	88 (9%)
Pulmonary disease	165 (11%)	6 (6%)	31 (14%)	27 (11%)	101 (10%)
Total prescription medications (*n*)*	2.0 (1.0–4.0)	3.0 (1.0–5.0)	3.0 (1.0–4.0)	3.0 (1.0–5.0)	2.0 (1.0–4.0)

*Note*: Weight change was defined as >3% annual weight change from Years 2 to 3 and to year 4 visits.

* *p* < 0.05.

**FIGURE 1:**
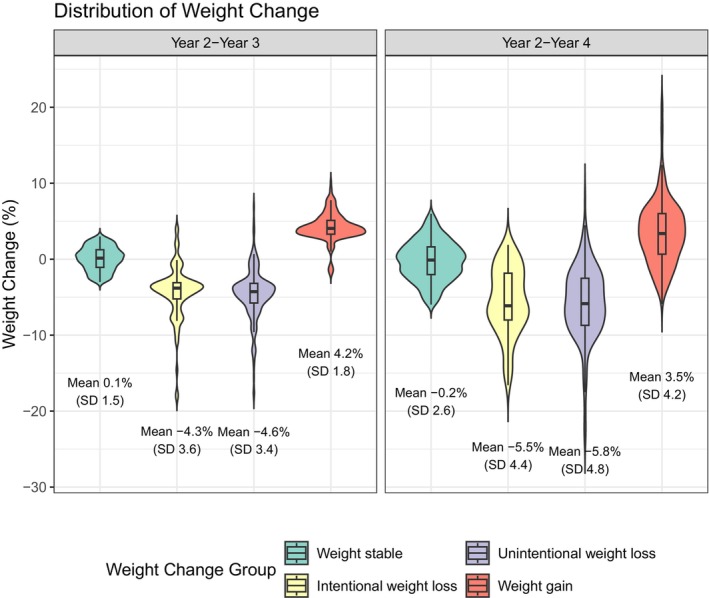
Distribution of weight change among Health ABC participants by weight change groups (*N* = 1536).

On average, those with unintentional weight loss lost 4.6%, on average, of body weight from Years 2 to 3 and 5.8% from Years 2 to 4, followed by 4.3% and 5.5% in the intentional weight loss group. The weight stable group gained 0.1% from Years 2 to 3 but lost 0.2% from Years 2 to 4. The weight gain group showed increases of 4.2% and 3.5% body weight from Years 2 to 4, respectively. (Figure 1).

**FIGURE 2:**
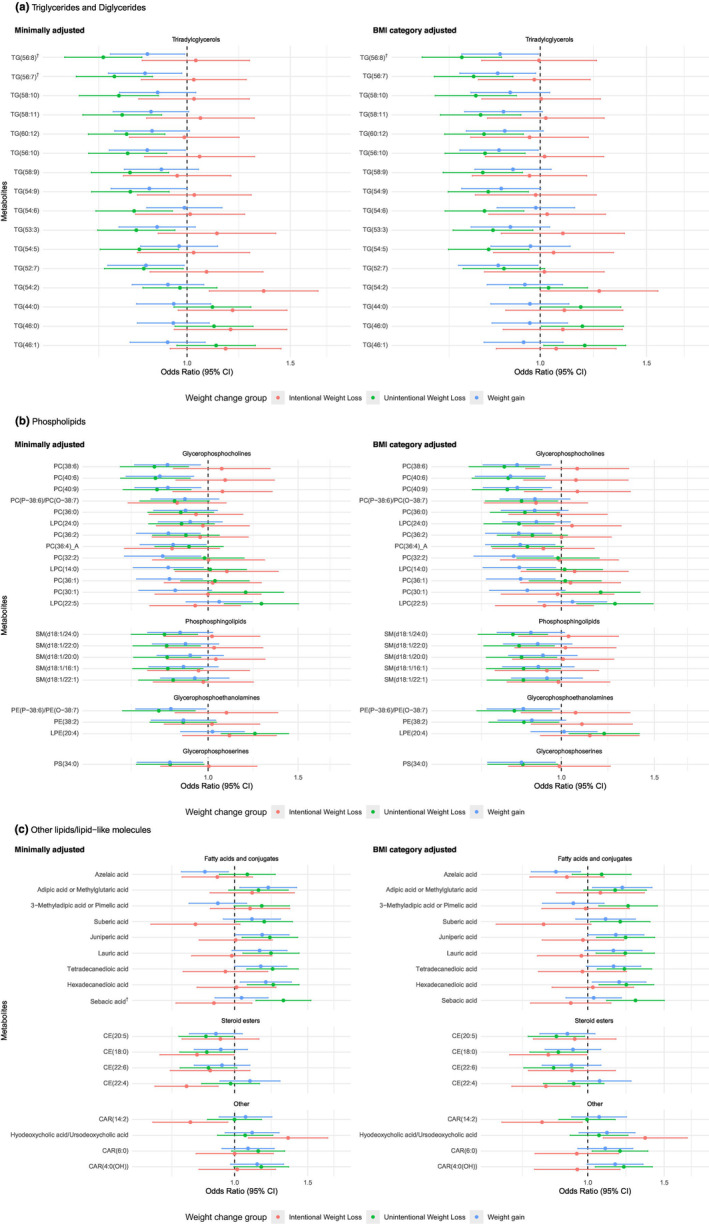
(A) Significant associations (*p* < 0.05) between triglycerides (per one standard deviation of log‐transformed values) and weight change groups from multinomial logistic regression adjusting for age, sex, race, and BMI category in Health ABC participants (*N* = 1536) (B) Significant associations (*p* < 0.05) between phospholipids (per one standard deviation of log‐transformed values) and weight change groups from multinomial logistic regression adjusting for age, sex, race (minimally adjusted), and BMI category in Health ABC participants (*N* = 1536) (C) Significant associations (*p* < 0.05) between other lipids and lipid‐like molecules (per one standard deviation of log‐transformed values) and weight change groups from multinomial logistic regression adjusting for age, sex, race (minimally adjusted), and BMI category in Health ABC participants (*N* = 1536) Metabolites were organized by Human Metabolome Database Taxonomy sub class and the odds ratios for unintentional weight loss. † represents metabolites significantly associated with unintentional weight loss relative to weight stable after multiple comparison adjustment (FDR < 0.05).

**FIGURE 3:**
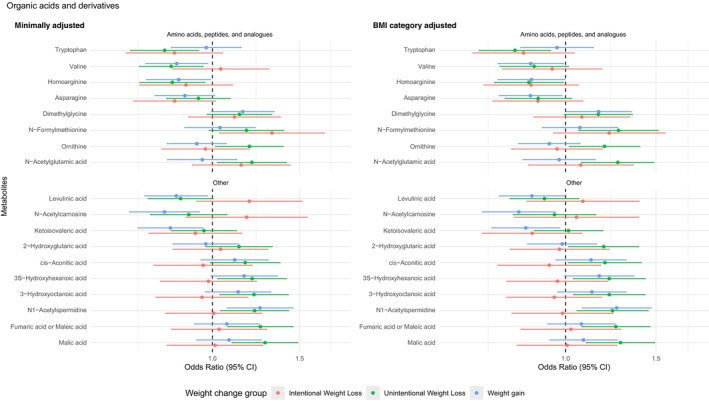
Significant associations (*p* < 0.05) of amino acids/peptides and analogs and other organic acids (per one standard deviation of log‐transformed values) with weight change groups from multinomial logistic regression adjusting for age, sex, race (minimally adjusted), and BMI category in Health ABC participants (*N* = 1536) Metabolites were organized by Human Metabolome Database Taxonomy sub class and the odds ratios for unintentional weight loss.

**FIGURE 4:**
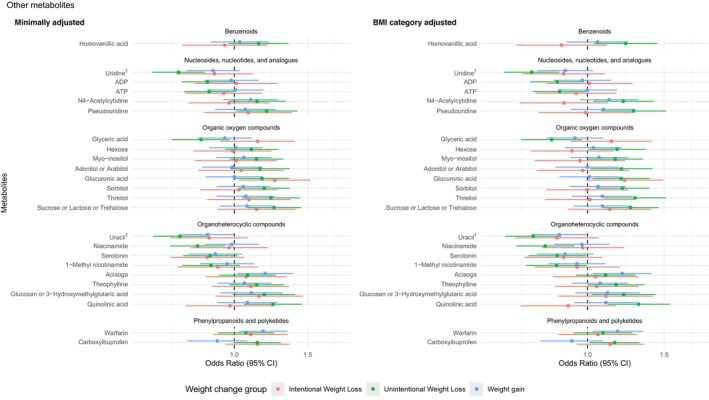
Significant associations (*p* < 0.05) of remaining metabolites (per one standard deviation of log‐transformed values) with weight change groups from multinomial logistic regression adjusting for age, sex, race (minimally adjusted), and BMI category in Health ABC participants (*N* = 1536) Metabolites were organized by Human Metabolome Database Taxonomy sub class and the odds ratios for unintentional weight loss. † represents metabolites significantly associated with unintentional weight loss relative to weight stable after multiple comparison adjustment (FDR < 0.05).

Out of 442 unique metabolites analyzed, 109 were associated with weight change groups (*p* < 0.05) after adjusting for age, race, and sex, and 5 remained significant after adjusting for multiple comparisons (FDR < 0.05). After further adjusting for BMI categories, 97 unique metabolites were significantly associated with weight change groups (*p* < 0.05), including 5 with intentional weight loss, 77 with unintentional weight loss, and 30 with weight gain when compared with weight stable participants. (Figures 2, 3, 4) There was no statistically significant non‐linear term for any metabolites after adjusting for multiple comparisons (all FDRs > 0.05). There was no statistically significant interaction between the metabolites and race, sex, and BMI categories after adjusting for multiple comparisons (all FDRs > 0.05). Subgroup analyses for the 97 metabolites across race and sex, as well as across BMI categories, are presented in Tables S3 and S4.

Among the 77 metabolites significantly associated with unintentional weight loss (*p* < 0.05), higher levels of 7 fatty acids, 2 acyl‐carnitines, 3 phospholipids, 3 triglycerides (TG(44:0), TG(46:0), and TG(46:1)), 3 amino acid derivatives, 7 other organic acids, 7 organic oxygen compounds (sugars), and 7 other organic compounds were linked to higher odds of unintentional weight loss. Conversely, lower levels of 11 triglycerides with more double bonds (majority ≥7), 6 phosphatidylcholines and plasmalogens, 5 phosphosphingolipids, 2 phosphoethanolamine and plasmalogens, 1 phosphoserine, 3 cholesterol esters, 2 amino acids, 3 nucleosides/nucleotides and analogs (uridine, ADP, ATP), and 5 other organic compounds were linked to higher odds of unintentional weight loss. Only three metabolites, including TG(56:8), uridine, and uracil, remained significant after multiple comparison adjustment when compared with weight stable (FDR < 0.05).

Furthermore, one triglycerides (TG(54:2)) and one bile acid (ursodeoxycholic acid) were associated with higher odds of intentional weight loss, while lower levels of two cholesterol esters and one acyl‐carnitine were associated with higher odds of intentional weight loss compared with weight stable (*p* < 0.05). For weight gain, higher levels of three medium‐to‐long chain fatty acids, one amino acid (dimethylglycine), and one other organic acid (N1‐acetylspermidine), and two other organic compounds were linked to higher odds of weight gain, while lower levels of five triglycerides with ≥7 double bonds, ten phospholipids, one medium‐chain saturated fatty acid (azelaic acid), three amino acids, three other organic acids, and uracil were linked to higher odds of weight gain (*p* < 0.05). No metabolite associations for intentional weight loss or weight gain remained significant after adjusting for multiple comparisons.

**FIGURE 5:**
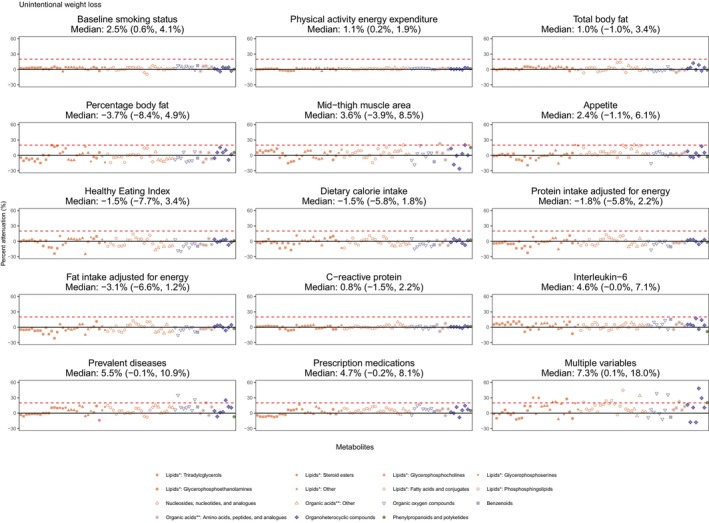
Percent attenuation in the association between a metabolite and unintentional weight loss relative to weight stable after adjusting for potential confounding factor(s) in addition to age, sex, race, and BMI category among 77 significant metabolites (*p* < 0.05) Metabolite were organized by the Human Metabolome Database taxonomy super class and subclass. Percent attenuation = 100*(*β*1 − *β*2)/*β*1, where *β*1 is the age, race, sex, and obesity‐adjusted beta coefficient between weight change and metabolite values and *β*2 is the beta coefficient after further adjusting for other important risk factor(s) specified on each subplot. Smoking, energy expenditure, total body fat, percentage body fat, mid‐thigh muscle area, Healthy Eating Index, total calorie intake, protein and fat intake adjusted for energy, C‐reactive protein, interleukin‐6, prevalent diseases and prescription medication were additionally adjusted together in the multiple variable adjusted model. The asterisk (*) represents lipids and lipid‐like molecules. The asterisks (**) represent organic acids and derivatives. Medians and interquartile ranges for percent attenuations across all metabolites are presented.

Figure 5 illustrates the percent attenuation in associations between each of the 77 single metabolites and unintentional weight loss compared with weight stable after adjusting for additional potential confounders. Most single risk factors, except for mid‐thigh muscle area, prevalent/history of diseases, and appetite, attenuated less than 20% of the metabolite associations of unintentional weight loss compared with weight stable. Prevalent or history of diseases attenuated half of the metabolite associations by >5.5% and particularly the association for hexose by 35%, myo‐inositol by 26%, theophylline by 25%, and N‐formylmethionine by 20%. Specifically, diabetes alone attenuated the association between hexose and unintentional weight loss by 30%. Lower mid‐thigh muscle area attenuated associations of glucosan and homoarginine with unintentional weight loss by 20% and 22%, respectively. The percent attenuation due to adjusting for lower muscle mass was greater than that due to total fat mass for majority of the metabolites including triglycerides, most phospholipids, organic acids such as tryptophan, and ADP. Poorer appetite attenuated associations between two metabolites and unintentional weight loss by 20%–21%, including CAR(6:0) and 3S‐hydrocyhexanoic acid. Compared with adjusting for dietary risk factors and physical activity, the percent attenuation for most metabolites (*n* > 40) were greater when adjusting for appetite. The fully adjusted model showed that 16 metabolite associations were attenuated by 20%–48%, including 2 cholesterol esters, 5 phospholipids, carboxyibuprofen, N‐formylmethionine, theophylline, glucosan, glucuronic acid, myo‐inositol, suberic acid, N1‐acetylspermidine, and N4‐acetylcytidine. Forty‐seven metabolites remained significantly associated with unintentional weight loss in the fully adjusted model.

## 4 DISCUSSION

In this biracial older cohort, we observed distinct metabolomic patterns associated with subsequent unintentional weight loss, intentional weight loss, and weight gain over 2 years, highlighting a need to differentiate between intentional and unintentional weight loss in older adults. The metabolites associated with unintentional weight loss were implicated in amino acid metabolism, mitochondrial dysfunction, and inflammation, which were also related to insulin resistance, sarcopenia, and composite healthy aging (Guasch‐Ferré et al., 2016; Long et al., 2020; Marron et al., 2019, 2024). While prevalent diseases, lower muscle mass, poorer appetite, and the combination of lifestyle and health condition risk factors explained part of these associations, the majority remained robust and independent of the potential confounders included.

Many lipids and lipid‐like molecules showed varied associations with different weight changes. For example, triacylglycerols (TAGs) with more double bonds were associated with lower odds of unintentional weight loss only, while TAGs with fewer double bonds were associated with higher odds of intentional weight loss only. This distinction highlights the need to distinguish between intentional and unintentional weight loss among older adults to avoid reverse causality in observational studies. In the Framingham Offspring study, studies have found higher levels of several TAGs with fewer double bonds associated with higher BMI cross‐sectionally (Ho et al., 2016) but more weight loss (Geidenstam et al., 2019), whereas TAGs with more double bonds associated with lower BMI (Ho et al., 2016) but less weight loss (Geidenstam et al., 2019). This suggests that the positive relationship between certain TAGs and intentional weight loss could be confounded by underlying indications (e.g., high BMI‐related health conditions) that drive older adults to lose weight intentionally. A weight control intention might mask the true metabolic mechanisms underlying weight regulation of older populations in observational, particularly cross‐sectional but also longitudinal, studies.

Higher levels of TAGs with more carbon and double bonds were associated with a lower odds of unintentional weight loss, which remain significant after adjusting for important risk factors. This finding is consistent with previous findings that TAGs with more double bonds were associated with more weight gain in adults (Geidenstam et al., 2019). TAGs with more double bond content might help maintain a stable weight in older adults not intended to lose weight. TAGs of higher carbon number and double bond content were associated with lower risk of diabetes (Rhee et al., 2011). In addition, during oral glucose tolerance testing, pharmacologic interventions, and acute exercise testing, these TAGs increased in response to insulin action and were poorly correlated with insulin resistance (Rhee et al., 2011). Therefore, higher concentrations of selected plasma TAGs might be indictive of lower risks of both metabolic disorders related to insulin resistance and unintentional weight loss in older adults. On the other hand, unintentional weight loss in older adults might have a metabolic dysregulation underpinning which might be subclinical and might not be detectable by clinical biomarkers such as hemoglobin A1c and fasting glucose. Lower levels of selected circulating TAGs might help identify older adults at risk for metabolic and weight changes.

Higher levels of several phospholipids and derivatized lipids (sphingomyelins, phosphatidylcholines, phosphatidylethanolamine plasmalogens) were generally associated with lower odds of unintentional weight loss, particularly those of longer carbon‐chain. Supporting our findings, higher levels of most PCs with more double bonds were associated with lower risk of diabetes while higher levels of some other PCs were associated with higher risk of diabetes in adults (Floegel et al., 2013). This again emphasized the significant metabolic alteration underlying unintentional weight loss. In addition, among all significant metabolites, we observed the greater percent attenuation after further adjustment of pro‐inflammation markers in three phosphatidylcholines (PC(40:6), PC(40:9), and PC(38:6)) than other metabolites, supporting an inflammation pathway of unintentional weight loss. Several phospholipid species have been considered as modulators of inflammation and apoptosis (Bozelli Jr. et al., 2021; Gonzalez‐Covarrubias, 2013; Wallner et al., 2018). In addition, phospholipids might also protect against oxidative stress (Johnson & Stolzing, 2019). These results underscore the importance of chronic inflammation in biological and maybe pathological aging (*inflammaging*) (Ferrucci & Fabbri, 2018; Franceschi et al., 2017, 2018).

Interestingly, we observed negative associations of branched‐chain amino acids (BCAAs) and aromatic amino acids (AAAs) with unintentional weight loss, although some were borderline significant, even after adjusting for protein intake, contrary to findings in obesity (Ahmad et al., 2022), weight gain (Wahl et al., 2015) and diabetes (Guasch‐Ferré et al., 2016; Long et al., 2020) among adults but is consistent with those for sarcopenia in older individuals (Lu et al., 2020). BCAAs and AAAs are crucial for protein synthesis and energy production with BCAAs also inhibiting protein catabolism. Our observations suggest that muscle wasting may play a critical role in unintentional weight loss and its related adverse outcomes. While insulin can significantly increase muscle protein synthesis in young adults when muscle amino acid availability is increased (Fujita & Volpi, 2006), healthy, nondiabetic older humans exhibit resistance, requiring supraphysiological hyperinsulinemia for stimulation (Fujita et al., 2009; Fujita & Volpi, 2006). Despite this resistance, older muscles still respond to amino acids, particularly BCAAs, which stimulate muscle protein synthesis in older adults (Fujita & Volpi, 2006). Therefore, despite the potential muscle wasting effect of insulin resistance with aging, higher circulating amino acids might help reduce the risk of sarcopenia in older adults.

To summarize, the observed metabolite correlates underscore the importance of amino acids and lipids metabolism in subsequent unintentional weight loss. Subclinical metabolic change and muscle wasting related to inflammation, energy metabolism, and protein catabolism may be the key to understand unintentional weight loss in older adults. Indeed, lower amino acids including BCAAs and AAAs, and lower phospholipids were also related to frailty and its energy level and weight loss component in older adults (Marron et al., 2019, 2024). Moreover, the observed negative associations between ADP and ATP with unintentional weight loss also suggest that a deficiency in energy production might contribute to unintentional weight loss and frailty in older adults. These findings, taken together, may indicate a critical role of mitochondrial dysfunction in unintentional weight loss and its related health outcomes among older adults. Supporting this notion, previous studies have found that TAGs, phospholipids, and BCAAs were significantly associated with mitochondrial function in animal models and human research (Aflaki et al., 2011; Vue et al., 2023; Zhenyukh et al., 2018).

We found that prevalent diseases explained many metabolite associations by at least 5%, suggesting a role of diseases such as diabetes underlying unintentional weight loss. It is also worth noting that lower baseline muscle mass showed a more substantial impact on observed metabolite associations than body fat did, both on average and particularly for certain metabolites. This, again, emphasized a potentially critical role of low muscle mass, which can result from muscle wasting, in the metabolism and unintentional weight loss pathways. Additionally, poorer appetite also exhibited a more substantial impact on most metabolite associations with unintentional weight loss than dietary quality, dietary intake, and physical activity level. Poor appetite might be either a confounding factor or a mediator in the metabolism‐unintentional weight loss pathways. Loss of appetite or so‐called anorexia, regardless of cause, is a significant risk factor for unexplained weight loss in older adults (McMinn et al., 2011). In our study, poorer appetite was more common, while the calorie intake was higher and the energy expenditure by physical activity was lower, in older adults who subsequently lost weight unintentionally than in those who intended to lose weight. These potentially indicate a complex but unknown interplay between metabolism, appetite, and unintentional weight loss, which might be independent of dietary intake and physical activity. However, despite their lack of impact on unintentional weight loss‐related metabolite associations, this does not rule out the potential beneficial effect of diet and exercise intervention in preventing sarcopenia during unintentional weight loss. Although most individual risk factors did not have a pronounced impact on the observed metabolite associations, the combination of these risk factors did affect 14 metabolites. This suggests that many metabolite associations with unintentional weight loss and their related pathways may be attributed to known risk factors. Nevertheless, the majority of the observed metabolite associations with unintentional weight loss remained robust against potential lifestyle and health conditions observed before the weight change occurred.

Strengths of our study included a well‐characterized, biracial older population with prospective assessment of intention before repeated body weight, and a detailed lifestyle and metabolic quantification, enabling the interrogation of the pathways of unintentional weight loss. Our prospective design provides a piece of temporal evidence that allows us to speculate the direction of a potential causal relationship. However, we are not able to rule out the possibility of reverse causality or bi‐directional relationship. In addition, our findings need to be interpreted with consideration of some limitations. First, the generalizability of our results was limited by the relatively healthy study population. Future studies in older adults across a wider functional range are warranted to validate our findings. Second, weight loss intentions might have changed over time. Using baseline intention to extrapolate participants' intention during a 2‐year period might underestimate the metabolite associations of unintentional/intentional weight loss. Third, like other observational studies, despite our effort to control for potential confounders, residual confounding might exist. Last, no easy method exists to comprehensively measure circulating metabolites such as TAGs with varied carbon and double bond contents, thus the application of the findings is currently limited.

In conclusion, we identified metabolic signatures of incident unintentional weight loss distinct from intentional weight change in a large group of White and Black older Americans. These metabolic characterizations involve lipid metabolism and amino acid metabolism pathways related to mitochondrial dysfunction, oxidative stress, protein catabolism and inflammation. The combination of known lifestyle risk factors and health conditions can account for part of these metabolic profiles, with chronic diseases, low muscle mass and poor appetite appearing to play a more prominent role in at least some metabolic mechanisms of unintentional weight loss. Future investigations of metabolic function in related pathways, muscle loss, poor appetite, and weight regulation in older populations are warranted to validate our results.

## AUTHOR CONTRIBUTIONS

SSY conceptualized the analysis, analyzed data, constructed illustrations and wrote the manuscript. MMM, SF, IM, GCT, RVS & VLM discussed, carefully reviewed and revised manuscript. ABN conceptualized the study and analysis, reviewed and revised the manuscript. All other coauthors carefully reviewed and revised the manuscript.

## FUNDING INFORMATION

This work was supported by National Institute on Aging R01‐AG‐059729. The Health ABC cohort was supported by National Institute on Aging Contracts N01‐AG‐6‐2101, N01‐AG‐6‐2103, and N01‐AG‐6‐2106; National Institute on Aging Grant R01‐AG028050, and National Institute of Nursing Research Grant R01‐NR012459.

## CONFLICT OF INTEREST STATEMENT

In the past 12 months, Shah has served for a consultant for Amgen, Cytokinetics, and Thryv Therapeutics (with options ownership in Thryv). Shah is a co‐inventor on a patent for ex‐RNAs signatures of cardiac remodeling and a pending patent on proteomic signatures of fitness and lung and liver diseases. Murthy owns stock in General Electric, Cardinal Health, Pfizer, Amgen, Merck, Viatris and Johnson & Johnson and stock options in Ionetix. He is a paid consultant for INVIA Medical Imaging Solutions & Siemens Healthineers. Murthy has received research support through his institution from Siemens Healthineers. Murthy is supported by the Melvyn Rubenfire Professorship in Preventive Cardiology. Murthy and Shah are also supported partly by grants from the National Institutes of Health and American Heart Association. Marron is supported by the National Institute on Aging K01‐AG‐075143. Newman is supported by the Pittsburgh Pepper Center P30‐AG024827. Farsijani is supported by a career development award from the National Institute on Aging (K01 AG071855).

## Supporting information


Table S1.

## Data Availability

Data supporting this study are available from https://healthabc.nia.nih.gov/.

## References

[acel14410-bib-0001] Aflaki, E. , Radovic, B. , Chandak, P. G. , Kolb, D. , Eisenberg, T. , Ring, J. , Fertschai, I. , Uellen, A. , Wolinski, H. , Kohlwein, S. D. , Zechner, R. , Levak‐Frank, S. , Sattler, W. , Graier, W. F. , Malli, R. , Madeo, F. , & Kratky, D. (2011). Triacylglycerol accumulation activates the mitochondrial apoptosis pathway in macrophages. The Journal of Biological Chemistry, 286, 7418–7428. 21196579 10.1074/jbc.M110.175703 PMC3044998

[acel14410-bib-0002] Ahmad, S. , Hammar, U. , Kennedy, B. , Salihovic, S. , Ganna, A. , Lind, L. , Sundström, J. , Ärnlöv, J. , Berne, C. , Risérus, U. , Magnusson, P. K. E. , Larsson, S. C. , & Fall, T. (2022). Effect of general adiposity and central body fat distribution on the circulating metabolome: A multicohort nontargeted metabolomics observational and Mendelian randomization study. Diabetes, 71, 329–339. 34785567 10.2337/db20-1120

[acel14410-bib-0003] Albert, S. M. , Venditti, E. M. , Boudreau, R. M. , Kieffer, L. A. , Rager, J. R. , Zgibor, J. C. , Vander Bilt, J. , Danielson, M. E. , Burke, L. E. , Glynn, N. W. , Jakicic, J. M. , Smith, K. J. , Semler, L. N. , & Newman, A. B. (2022). Weight loss through lifestyle intervention improves mobility in older adults. Gerontologist, 62, 931–941. 33822933 10.1093/geront/gnab048 PMC9653001

[acel14410-bib-0004] Almanza‐Aguilera, E. , Brunius, C. , Bernal‐Lopez, M. R. , Garcia‐Aloy, M. , Madrid‐Gambin, F. , Tinahones, F. J. , Gómez‐Huelgas, R. , Landberg, R. , & Andres‐Lacueva, C. (2018). Impact in plasma metabolome as effect of lifestyle intervention for weight‐loss reveals metabolic benefits in metabolically healthy obese women. Journal of Proteome Research, 17, 2600–2610. 29877711 10.1021/acs.jproteome.8b00042

[acel14410-bib-0005] Benjamini, Y. , & Hochberg, Y. (1995). Controlling the false discovery rate: A practical and powerful approach to multiple testing. Journal of the Royal Statistical Society: Series B: Methodological, 57, 289–300.

[acel14410-bib-0006] Bihlmeyer, N. A. , Kwee, L. C. , Clish, C. B. , Deik, A. A. , Gerszten, R. E. , Pagidipati, N. J. , Laferrère, B. , Svetkey, L. P. , Newgard, C. B. , Kraus, W. E. , & Shah, S. H. (2021). Metabolomic profiling identifies complex lipid species and amino acid analogues associated with response to weight loss interventions. PLoS One, 16, e0240764. 34043632 10.1371/journal.pone.0240764 PMC8158886

[acel14410-bib-0007] Bozelli, J. C., Jr. , Azher, S. , & Epand, R. M. (2021). Plasmalogens and chronic inflammatory diseases. Frontiers in Physiology, 12, 730829. 34744771 10.3389/fphys.2021.730829 PMC8566352

[acel14410-bib-0008] Cao, Y. , Hardy, R. , & Wulaningsih, W. (2019). Associations of medical conditions, lifestyle and unintentional weight loss in early old age: The 1946 British birth cohort. PLoS One, 14, e0211952. 30964855 10.1371/journal.pone.0211952 PMC6456161

[acel14410-bib-0009] Cesari, M. , Penninx, B. W. , Newman, A. B. , Kritchevsky, S. B. , Nicklas, B. J. , Sutton‐Tyrrell, K. , Rubin, S. M. , Ding, J. , Simonsick, E. M. , Harris, T. B. , & Pahor, M. (2003). Inflammatory markers and onset of cardiovascular events: Results from the health ABC study. Circulation, 108, 2317–2322. 14568895 10.1161/01.CIR.0000097109.90783.FC

[acel14410-bib-0010] de Souto, B. P. (2022). Editorial: Poor appetite and aging: The role of physical activity under a geroscience perspective. Journal of Nutrition, Health & Aging, 26, 907–908.10.1007/s12603-022-1849-x36259578

[acel14410-bib-0011] de Stefani, F. D. C. , Pietraroia, P. S. , Fernandes‐Silva, M. M. , Faria‐Neto, J. , & Baena, C. P. (2018). Observational evidence for unintentional weight loss in all‐cause mortality and major cardiovascular events: A systematic review and meta‐analysis. Scientific Reports, 8, 15447. 30337578 10.1038/s41598-018-33563-z PMC6194006

[acel14410-bib-0012] Deravi, N. , Moazzeni, S. S. , Hasheminia, M. , Hizomi Arani, R. , Azizi, F. , & Hadaegh, F. (2022). Three‐year weight change and risk of all‐cause, cardiovascular, and cancer mortality among Iranian adults: Over a decade of follow‐up in the Tehran lipid and glucose study. BMC Public Health, 22, 1762. 36114528 10.1186/s12889-022-14126-4 PMC9482273

[acel14410-bib-0013] Economos, C. D. , Nelson, M. E. , Fiatarone, M. A. , Dallal, G. E. , Heymsfield, S. B. , Wang, J. , Yasumara, S. , Ma, R. , Vaswani, A. N. , Russell‐Aulet, M. , & Pierson, R. N. (1997). A multi‐center comparison of dual energy X‐ray absorptiometers: In vivo and in vitro soft tissue measurement. European Journal of Clinical Nutrition, 51, 312–317. 9152682 10.1038/sj.ejcn.1600400

[acel14410-bib-0014] Ferrucci, L. , & Fabbri, E. (2018). Inflammageing: Chronic inflammation in ageing, cardiovascular disease, and frailty. Nature Reviews. Cardiology, 15, 505–522. 30065258 10.1038/s41569-018-0064-2 PMC6146930

[acel14410-bib-0015] Figueroa‐Colon, R. , Mayo, M. S. , Treuth, M. S. , Aldridge, R. A. , & Weinsier, R. L. (1998). Reproducibility of dual‐energy X‐ray absorptiometry measurements in prepubertal girls. Obesity Research, 6, 262–267. 9688102 10.1002/j.1550-8528.1998.tb00348.x

[acel14410-bib-0016] Floegel, A. , Stefan, N. , Yu, Z. , Mühlenbruch, K. , Drogan, D. , Joost, H. G. , Fritsche, A. , Häring, H. U. , Hrabě de Angelis, M. , Peters, A. , Roden, M. , Prehn, C. , Wang‐Sattler, R. , Illig, T. , Schulze, M. B. , Adamski, J. , Boeing, H. , & Pischon, T. (2013). Identification of serum metabolites associated with risk of type 2 diabetes using a targeted metabolomic approach. Diabetes, 62, 639–648. 23043162 10.2337/db12-0495 PMC3554384

[acel14410-bib-0017] Franceschi, C. , Garagnani, P. , Parini, P. , Giuliani, C. , & Santoro, A. (2018). Inflammaging: A new immune‐metabolic viewpoint for age‐related diseases. Nature Reviews. Endocrinology, 14, 576–590. 10.1038/s41574-018-0059-4 30046148

[acel14410-bib-0018] Franceschi, C. , Garagnani, P. , Vitale, G. , Capri, M. , & Salvioli, S. (2017). Inflammaging and 'garb‐aging'. Trends in Endocrinology and Metabolism, 28, 199–212. 27789101 10.1016/j.tem.2016.09.005

[acel14410-bib-0019] Fujita, S. , Glynn, E. L. , Timmerman, K. L. , Rasmussen, B. B. , & Volpi, E. (2009). Supraphysiological hyperinsulinaemia is necessary to stimulate skeletal muscle protein anabolism in older adults: Evidence of a true age‐related insulin resistance of muscle protein metabolism. Diabetologia, 52, 1889–1898. 19588121 10.1007/s00125-009-1430-8 PMC2843438

[acel14410-bib-0020] Fujita, S. , & Volpi, E. (2006). Amino acids and muscle loss with aging. The Journal of Nutrition, 136, 277S–280S. 16365098 10.1093/jn/136.1.277S PMC3183816

[acel14410-bib-0021] Fuller, N. J. , Laskey, M. A. , & Elia, M. (1992). Assessment of the composition of major body regions by dual‐energy X‐ray absorptiometry (DEXA), with special reference to limb muscle mass. Clinical Physiology, 12, 253–266. 1606809 10.1111/j.1475-097x.1992.tb00831.x

[acel14410-bib-0022] Geidenstam, N. , Hsu, Y. H. , Astley, C. M. , Mercader, J. M. , Ridderstråle, M. , Gonzalez, M. E. , Gonzalez, C. , Hirschhorn, J. N. , & Salem, R. M. (2019). Using metabolite profiling to construct and validate a metabolite risk score for predicting future weight gain. PLoS One, 14, e0222445. 31560688 10.1371/journal.pone.0222445 PMC6764659

[acel14410-bib-0023] Gonzalez‐Covarrubias, V. (2013). Lipidomics in longevity and healthy aging. Biogerontology, 14, 663–672. 23948799 10.1007/s10522-013-9450-7

[acel14410-bib-0024] Guasch‐Ferré, M. , Hruby, A. , Toledo, E. , Clish, C. B. , Martínez‐González, M. A. , Salas‐Salvadó, J. , & Hu, F. B. (2016). Metabolomics in prediabetes and diabetes: A systematic review and meta‐analysis. Diabetes Care, 39, 833–846. 27208380 10.2337/dc15-2251 PMC4839172

[acel14410-bib-0025] Guo, J. , Marseglia, A. , Shang, Y. , Dove, A. , Grande, G. , Fratiglioni, L. , & Xu, W. (2023). Association between late‐life weight change and dementia: A population‐based cohort study. Journals of Gerontology. Series A, Biological Sciences and Medical Sciences, 78, 143–150. 35921193 10.1093/gerona/glac157 PMC9879755

[acel14410-bib-0026] Haugsgjerd, T. R. , Dierkes, J. , Vollset, S. E. , Vinknes, K. J. , Nygård, O. K. , Seifert, R. , Sulo, G. , & Tell, G. S. (2017). Association between weight change and mortality in community living older people followed for up to 14 years. The Hordaland Health Study (HUSK). Journal of Nutrition, Health & Aging, 21, 909–917.10.1007/s12603-016-0866-z28972244

[acel14410-bib-0027] Heianza, Y. , Sun, D. , Smith, S. R. , Bray, G. A. , Sacks, F. M. , & Qi, L. (2018). Changes in gut microbiota‐related metabolites and long‐term successful weight loss in response to weight‐loss diets: The POUNDS lost trial. Diabetes Care, 41, 413–419. 29305401 10.2337/dc17-2108 PMC5829970

[acel14410-bib-0028] Ho, J. E. , Larson, M. G. , Ghorbani, A. , Cheng, S. , Chen, M. H. , Keyes, M. , Rhee, E. P. , Clish, C. B. , Vasan, R. S. , Gerszten, R. E. , & Wang, T. J. (2016). Metabolomic profiles of body mass index in the framingham heart study reveal distinct cardiometabolic phenotypes. PLoS One, 11, e0148361. 26863521 10.1371/journal.pone.0148361 PMC4749349

[acel14410-bib-0029] Hubner, S. , Boron, J. B. , & Koehler, K. (2021). The effects of exercise on appetite in older adults: A systematic review and meta‐analysis. Frontiers in Nutrition, 8, 734267. 34869516 10.3389/fnut.2021.734267 PMC8638160

[acel14410-bib-0030] Johnson, A. A. , & Stolzing, A. (2019). The role of lipid metabolism in aging, lifespan regulation, and age‐related disease. Aging Cell, 18, e13048. 31560163 10.1111/acel.13048 PMC6826135

[acel14410-bib-0031] Lee, J. S. , Kritchevsky, S. B. , Tylavsky, F. A. , Harris, T. , Everhart, J. , Simonsick, E. M. , Rubin, S. M. , Newman, A. B. , & Health, Aging and Body Composition (Health ABC) Study . (2004). Weight‐loss intention in the well‐functioning, community‐dwelling elderly: Associations with diet quality, physical activity, and weight change. American Journal of Clinical Nutrition, 80, 466–474. 15277172 10.1093/ajcn/80.2.466

[acel14410-bib-0032] Long, J. , Yang, Z. , Wang, L. , Han, Y. , Peng, C. , Yan, C. , & Yan, D. (2020). Metabolite biomarkers of type 2 diabetes mellitus and pre‐diabetes: A systematic review and meta‐analysis. BMC Endocrine Disorders, 20, 174. 33228610 10.1186/s12902-020-00653-x PMC7685632

[acel14410-bib-0033] Lu, Y. , Karagounis, L. G. , Ng, T. P. , Carre, C. , Narang, V. , Wong, G. , Tan, C. T. Y. , Zin Nyunt, M. S. , Gao, Q. , Abel, B. , Poidinger, M. , Fulop, T. , Bosco, N. , & Larbi, A. (2020). Systemic and metabolic signature of sarcopenia in community‐dwelling older adults. Journals of Gerontology. Series A, Biological Sciences and Medical Sciences, 75, 309–317. 30624690 10.1093/gerona/glz001

[acel14410-bib-0034] Lynch, D. H. , Rushing, B. R. , Pathmasiri, W. , McRitchie, S. , Batchek, D. J. , Petersen, C. L. , Gross, D. C. , Sumner, S. C. J. , & Batsis, J. A. (2023). Baseline serum biomarkers predict response to a weight loss intervention in older adults with obesity: A pilot study. Metabolites, 13, 13. 10.3390/metabo13070853 PMC10385260 37512560

[acel14410-bib-0035] Marron, M. M. , Harris, T. B. , Boudreau, R. M. , Clish, C. B. , Moore, S. C. , Murphy, R. A. , Murthy, V. L. , Sanders, J. L. , Shah, R. V. , Tseng, G. C. , Wendell, S. G. , Zmuda, J. M. , & Newman, A. B. (2019). Metabolites associated with vigor to frailty among community‐dwelling older black men. Metabolites, 9, 9. 10.3390/metabo9050083 PMC6572139 31052232

[acel14410-bib-0036] Marron, M. M. , Yao, S. , Shah, R. V. , Murthy, V. L. , & Newman, A. B. (2024). Metabolomic characterization of vigor to frailty among community‐dwelling older black and white men and women. Geroscience, 46, 2371–2389. 37968423 10.1007/s11357-023-01005-y PMC10828147

[acel14410-bib-0037] McMinn, J. , Steel, C. , & Bowman, A. (2011). Investigation and management of unintentional weight loss in older adults. BMJ, 342, d1732. 21447571 10.1136/bmj.d1732

[acel14410-bib-0038] Newman, A. B. , Lee, J. S. , Visser, M. , Goodpaster, B. H. , Kritchevsky, S. B. , Tylavsky, F. A. , Nevitt, M. , & Harris, T. B. (2005). Weight change and the conservation of lean mass in old age: The health, aging and body composition study. American Journal of Clinical Nutrition, 82, 872–878. 16210719 10.1093/ajcn/82.4.872

[acel14410-bib-0039] Palmer, A. K. , & Jensen, M. D. (2022). Metabolic changes in aging humans: Current evidence and therapeutic strategies. The Journal of Clinical Investigation, 132, e158451. 35968789 10.1172/JCI158451 PMC9374375

[acel14410-bib-0040] Park, S. Y. , Wilkens, L. R. , Maskarinec, G. , Haiman, C. A. , Kolonel, L. N. , & Marchand, L. L. (2018). Weight change in older adults and mortality: The multiethnic cohort study. International Journal of Obesity, 42, 205–212. 28885999 10.1038/ijo.2017.188 PMC5803382

[acel14410-bib-0041] Perez‐Cornago, A. , Brennan, L. , Ibero‐Baraibar, I. , Hermsdorff, H. H. , O'Gorman, A. , Zulet, M. A. , & Martínez, J. A. (2014). Metabolomics identifies changes in fatty acid and amino acid profiles in serum of overweight older adults following a weight loss intervention. Journal of Physiology and Biochemistry, 70, 593–602. 24402878 10.1007/s13105-013-0311-2

[acel14410-bib-0042] Porter Starr, K. N. , Orenduff, M. , McDonald, S. R. , Mulder, H. , Sloane, R. , Pieper, C. F. , & Bales, C. W. (2019). Influence of weight reduction and enhanced protein intake on biomarkers of inflammation in older adults with obesity. Journal of Nutrition in Gerontology and Geriatrics, 38, 33–49. 30810500 10.1080/21551197.2018.1564200 PMC6447442

[acel14410-bib-0043] Quillen, E. E. , Beavers, D. P. , O'Brien Cox, A. , Furdui, C. M. , Lee, J. , Miller, R. M. , Wu, H. , & Beavers, K. M. (2020). Use of Metabolomic profiling to understand variability in adiposity changes following an intentional weight loss intervention in older adults. Nutrients, 12, 3188. 33086512 10.3390/nu12103188 PMC7603124

[acel14410-bib-0044] Rhee, E. P. , Cheng, S. , Larson, M. G. , Walford, G. A. , Lewis, G. D. , McCabe, E. , Yang, E. , Farrell, L. , Fox, C. S. , O'Donnell, C. J. , Carr, S. A. , Vasan, R. S. , Florez, J. C. , Clish, C. B. , Wang, T. J. , & Gerszten, R. E. (2011). Lipid profiling identifies a triacylglycerol signature of insulin resistance and improves diabetes prediction in humans. Journal of Clinical Investigation, 121, 1402–1411. 21403394 10.1172/JCI44442 PMC3069773

[acel14410-bib-0045] Sahyoun, N. R. , Serdula, M. K. , Galuska, D. A. , Zhang, X. L. , & Pamuk, E. R. (2004). The epidemiology of recent involuntary weight loss in the United States population. Journal of Nutrition, Health & Aging, 8, 510–517. 15543425

[acel14410-bib-0046] Santanasto, A. J. , Glynn, N. W. , Newman, M. A. , Taylor, C. A. , Brooks, M. M. , Goodpaster, B. H. , & Newman, A. B. (2011). Impact of weight loss on physical function with changes in strength, muscle mass, and muscle fat infiltration in overweight to moderately obese older adults: A randomized clinical trial. Journal of Obesity, 2011, 1–10. 10.1155/2011/516576 PMC2952914 20953373

[acel14410-bib-0047] Underland, L. J. , Schnatz, P. F. , Wild, R. A. , Saquib, N. , Shadyab, A. H. , Allison, M. , Banack, H. , & Wassertheil‐Smoller, S. (2022). The impact of weight change and measures of physical functioning on mortality. Journal of the American Geriatrics Society, 70, 1228–1235. 34988972 10.1111/jgs.17626 PMC8986581

[acel14410-bib-0048] Visser, M. , Goodpaster, B. H. , Kritchevsky, S. B. , Newman, A. B. , Nevitt, M. , Rubin, S. M. , Simonsick, E. M. , Harris, T. B. , & for the Health ABC Study . (2005). Muscle mass, muscle strength, and muscle fat infiltration as predictors of incident mobility limitations in well‐functioning older persons. Journals of Gerontology. Series A, Biological Sciences and Medical Sciences, 60, 324–333. 15860469 10.1093/gerona/60.3.324

[acel14410-bib-0049] Vue, Z. , Garza‐Lopez, E. , Neikirk, K. , Katti, P. , Vang, L. , Beasley, H. , Shao, J. , Marshall, A. G. , Crabtree, A. , Murphy, A. C. , Jenkins, B. C. , Prasad, P. , Evans, C. , Taylor, B. , Mungai, M. , Killion, M. , Stephens, D. , Christensen, T. A. , Lam, J. , … Hinton, A., Jr. (2023). 3D reconstruction of murine mitochondria reveals changes in structure during aging linked to the MICOS complex. Aging Cell, 22, e14009. 37960952 10.1111/acel.14009 PMC10726809

[acel14410-bib-0050] Wahl, S. , Vogt, S. , Stückler, F. , Krumsiek, J. , Bartel, J. , Kacprowski, T. , Schramm, K. , Carstensen, M. , Rathmann, W. , Roden, M. , Jourdan, C. , Kangas, A. J. , Soininen, P. , Ala‐Korpela, M. , Nöthlings, U. , Boeing, H. , Theis, F. J. , Meisinger, C. , Waldenberger, M. , … Grallert, H. (2015). Multi‐omic signature of body weight change: Results from a population‐based cohort study. BMC Medicine, 13, 48. 25857605 10.1186/s12916-015-0282-y PMC4367822

[acel14410-bib-0051] Wallner, S. , Orso, E. , Grandl, M. , Konovalova, T. , Liebisch, G. , & Schmitz, G. (2018). Phosphatidylcholine and phosphatidylethanolamine plasmalogens in lipid loaded human macrophages. PLoS One, 13, e0205706. 30308051 10.1371/journal.pone.0205706 PMC6181407

[acel14410-bib-0052] Wannamethee, S. G. , Shaper, A. G. , & Lennon, L. (2005). Reasons for intentional weight loss, unintentional weight loss, and mortality in older men. Archives of Internal Medicine, 165, 1035–1040. 15883243 10.1001/archinte.165.9.1035

[acel14410-bib-0053] Willett, W. C. , Howe, G. R. , & Kushi, L. H. (1997). Adjustment for total energy intake in epidemiologic studies. The American Journal of Clinical Nutrition, 65, 1220S–1228S. 9094926 10.1093/ajcn/65.4.1220S

[acel14410-bib-0054] Yao, S. , Colangelo, L. A. , Perry, A. S. , Marron, M. M. , Yaffe, K. , Sedaghat, S. , Lima, J. A. C. , Tian, Q. , Clish, C. B. , Newman, A. B. , Shah, R. V. , & Murthy, V. L. (2024). Implications of metabolism on multi‐systems healthy aging across the lifespan. Aging Cell, 23, e14090. 38287525 10.1111/acel.14090 PMC11019145

[acel14410-bib-0055] Zhenyukh, O. , González‐Amor, M. , Rodrigues‐Diez, R. R. , Esteban, V. , Ruiz‐Ortega, M. , Salaices, M. , Mas, S. , Briones, A. M. , & Egido, J. (2018). Branched‐chain amino acids promote endothelial dysfunction through increased reactive oxygen species generation and inflammation. Journal of Cellular and Molecular Medicine, 22, 4948–4962. 30063118 10.1111/jcmm.13759 PMC6156282

