# Supplementary material for: Metabolomic characterization of unintentional weight loss among community‐dwelling older Black and White men and women
Source: Aging Cell. 2024 Nov 15;24(3):e14410. doi: 10.1111/acel.14410 (PMC11896220; doi:10.1111/acel.14410)
Supplement: Supplementary file 1 — Table S1. [file ACEL-24-e14410-s001.docx]

**Supplementary files**

eTable 1. Characteristics of participants from the Health, Aging and Body Composition study by previous weight change Weight change was defined as >3% annual weight change from Year 1 to Year 2 visit.

| **Characteristic** | **Weight change from Baseline (Year 1) to Year 2** | | | |
| --- | --- | --- | --- | --- |
|  | **Overall**, N = 2,330 | **Weight gain**, N = 361 | **Weight loss**, N = 432 | **Weight stable**, N = 1,537 |
| **N (%), Mean (SD), or Median [Q1, Q3]** | | | | |
| **Baseline appetite** |  |  |  |  |
| *Very good* | 957 (42%) | 159 (45%) | 132 (31%) | 666 (44%) |
| *Good* | 857 (37%) | 131 (37%) | 154 (36%) | 572 (38%) |
| *Moderate to Very poor* | 486 (21%) | 67 (19%) | 139 (33%) | 280 (18%) |
| **Appetite change from Year 1 to Year 2** |  |  |  |  |
| *Improved* | 253 (11%) | 71 (20%) | 40 (9%) | 142 (9%) |
| *Similar* | 1,864 (81%) | 269 (75%) | 317 (75%) | 1,278 (84%) |
| *Worsen* | 185 (8%) | 17 (5%) | 68 (16%) | 100 (7%) |
| **Diet changed from Year 1 to Year 2 for better health** | 863 (38%) | 119 (33%) | 182 (43%) | 562 (37%) |
| **Age** | 74.6 (2.9) | 74.5 (2.9) | 74.7 (2.8) | 74.7 (2.9) |
| **Race,** Black | 866 (37%) | 144 (40%) | 189 (44%) | 533 (35%) |
| **Sex,** Men | 1,149 (49%) | 175 (48%) | 199 (46%) | 775 (50%) |
| **More than high school education** | 1,783 (77%) | 261 (72%) | 319 (74%) | 1,203 (78%) |
| **Baseline smoker** | 206 (9%) | 35 (10%) | 41 (9%) | 130 (8%) |
| **Baseline sleep, hours/night** | 6.9 (1.4) | 6.8 (1.4) | 6.9 (1.3) | 6.9 (1.3) |
| **BMI, kg/m^2^** | 27.3 (4.8) | 28.4 (5.3) | 26.2 (4.6) | 27.3 (4.7) |
| **BMI category** |  |  |  |  |
| *<25 kg/m^2^* | 786 (34%) | 94 (26%) | 184 (43%) | 508 (33%) |
| *25-30 kg/m^2^* | 974 (42%) | 149 (41%) | 172 (40%) | 653 (42%) |
| *>=30 kg/m^2^* | 570 (24%) | 118 (33%) | 76 (18%) | 376 (24%) |
| **Baseline total body fat (kg)** | 26.8 (8.7) | 26.4 (9.6) | 27.2 (8.6) | 26.7 (8.5) |
| **Baseline %body fat** | 35.0 (7.7) | 34.4 (8.2) | 35.5 (7.7) | 34.9 (7.6) |
| **Baseline mid-thigh muscle area (cm-sq)** | 223.4 (55.7) | 219.4 (55.6) | 221.8 (54.8) | 224.7 (55.9) |
| **Have illness interferes appetite** | 199 (9%) | 28 (8%) | 52 (12%) | 119 (8%) |
| **Healthy Eating Index score** | 69.7 (12.1) | 68.7 (12.4) | 68.3 (12.6) | 70.3 (11.9) |
| **Calories (Kcal/d)** | 1,753 [1,370-2,232] | 1,795 [1,394-2,276] | 1,724 [1,393-2,176] | 1,744 [1,355-2,250] |
| **Daily macronutrients intake adjusted for total calorie intake** | | |  |  |
| Protein (g/d) | 65.4 [58.5-74.5] | 65.1 [57.3-72.5] | 64.9 [58.3-73.2] | 65.5 [58.8-74.9] |
| Fat (g/d) | 71.0 [61.6-80.5] | 71.7 [61.9-81.6] | 72.2 [63.4-81.4] | 70.8 [61.4-79.9] |
| Carbohydrates (g/d) | 247.3 [226.0-270.7] | 248.7 [224.8-271.9] | 246.5 [223.4-270.3] | 247 [226.5-270.7] |
| **Energy expenditure (Kcal/kg/Week)** | 3.0 [0.3-9.4] | 3.0 [0.2-8.8] | 1.9 [0.1-7.5] | 3.3 [0.5-10.1] |
| **Interleukin-6 (pg/mL)** | 2.3 [1.5-3.9] | 2.5 [1.6-4.3] | 2.6 [1.5-4.3] | 2.3 [1.5-3.7] |
| **C-reactive protein (ug/mL)** | 2.8 [1.2-6.2] | 2.8 [1.1-5.9] | 2.7 [1.2-6.3] | 3.0 [1.2-6.2] |
| **Baseline diseases** |  |  |  |  |
| Cardiovascular disease | 598 (26%) | 93 (26%) | 102 (24%) | 403 (27%) |
| Hypertension | 1,154 (50%) | 183 (51%) | 233 (54%) | 738 (48%) |
| Diabetes | 342 (15%) | 55 (15%) | 85 (20%) | 202 (13%) |
| Cancer | 459 (20%) | 68 (19%) | 85 (20%) | 306 (20%) |
| Peripheral artery disease | 109 (5%) | 21 (6%) | 21 (5%) | 67 (4%) |
| Osteoarthritis | 235 (10%) | 38 (11%) | 41 (10%) | 156 (10%) |
| Depression | 221 (10%) | 48 (13%) | 41 (10%) | 132 (9%) |
| Pulmonary disease | 262 (11%) | 50 (14%) | 47 (11%) | 165 (11%) |
| **Total prescription medications** | 3.0 [1.0-5.0] | 3.0 [1.0-5.0] | 3.0 [1.0-5.0] | 2.0 [1.0-4.0] |
| **Annual weight change (kg)** | 0.68 (2.10) | 0.27 (2.28) | 1.49 (2.45) | 0.55 (1.89) |
| **Weight change group** |  |  |  |  |
| Intentional weight loss | 163 (7%) | 39 (11%) | 25 (6%) | 99 (6%) |
| Unintentional weight loss | 350 (15%) | 71 (20%) | 58 (13%) | 221 (14%) |
| Weight gain | 437 (19%) | 52 (14%) | 148 (34%) | 237 (15%) |
| Weight stable | 1,380 (59%) | 199 (55%) | 201 (47%) | 980 (64%) |

eTable 2. Blood biomarkers of participants from the Health, Aging, and Body Composition study by weight change groups Weight change was defined as >3% annual weight change from Year 2 to Year 3 and to Year 4 visits. Blood lipid profiles were measured in fasting plasma and other biomarkers were measured in fasting serum.

| **Characteristic** | **Weight change group** | | | | |
| --- | --- | --- | --- | --- | --- |
|  | **Overall** | **Intentional weight loss** | **Unintentional weight loss** | **Weight gain** | **Weight stable** |
| N | 1,536 | 99 | 220 | 237 | 980 |
|  | Mean (SD) or Median [IQR] or n (%) | | | | |
| Year 2 blood biomarkers |  |  |  |  |  |
| Total cholesterol (mg/dL) | 206 [181-230] | 205 [178-228] | 206 [180-234] | 203 [178-229] | 207 [182-229] |
| Fasting glucose (mg/dL) | 94 [87-106] | 95 [88-112] | 94 [86-114] | 96 [86-107] | 94 [87-105] |
| Baseline kidney function biomarkers | |  |  |  |  |
| eGFR (mL/Min/1.73m^2^) | 76.0 (18.4) | 73.2 (18.5) | 75.7 (21.1) | 75.4 (18.2) | 76.5 (17.8) |
| Cystatin C (mg/dL) | 0.98 [0.86-1.12] | 0.99 [0.89-1.17] | 1.00 [0.83-1.15] | 0.99 [0.86-1.14] | 0.98 [0.86-1.11] |
| Creatinine (mg/dL) | 1.00 [0.90-1.20] | 1.00 [0.90-1.20] | 1.00 [0.90-1.20] | 1.00 [0.90-1.10] | 1.00 [0.90-1.18] |
| Baseline lipid biomarkers | |  |  |  |  |
| Total triglycerides (mg/dL) | 126.0 [90-169] | 131 [94-174] | 114 [83-160] | 120 [90-178] | 123 [92-168] |
| Total cholesterol (mg/dL) | 202 [178-229] | 207 [177-230] | 198 [175-233] | 201 [175-230] | 202 [179-227] |
| HDL (mg/dL) | 51 [42-62] | 47 [40-61] | 52 [42-64] | 51 [41-63] | 51 [43-61] |
| LDL (mg/dL) | 121 [100-143] | 122 [100-151] | 120 [96-146] | 122 [100-143] | 121 [100-142] |
| Baseline leptin (ng/dL) | 10 [5-20] | 13 [7-26] | 9 [5-17] | 11 [5-18] | 10 [5-20] |
| Baseline fasting glucose (mg/dL) | 94 [87-104] | 95 [88-108] | 94 [86-110] | 93 [87-104] | 94 [88-102] |
| Baseline hemoglobin A1C (%) | 6.10 [5.70-6.50] | 6.10 [5.70-6.68] | 6.20 [5.70-6.80] | 6.10 [5.70-6.60] | 6.00 [5.70-6.50] |

**eTable 3. Associations between metabolites and weight change groups in Black men, Black women, White men, and White women from the Health ABC study (N = 1536)** *Statistically significant interaction between metabolite and race after adjusting for age and BMI category but not multiple comparisons (p<0.05); ^¶^ Statistically significant interaction between metabolite and sex after adjusting for age and BMI category but not multiple comparisons (p<0.05). There was no significant interaction after adjusting for multiple comparisons (all FDRs>0.05).

| **Metabolites** | **HMDB ID** | **Black men** | | |  | **Black women** | | |  | **White men** | | |  | **White women** | | |
| --- | --- | --- | --- | --- | --- | --- | --- | --- | --- | --- | --- | --- | --- | --- | --- | --- |
|  |  | **Intentional weight loss** | **Unintentional weight loss** | **Weight gain** |  | **Intentional weight loss** | **Unintentional weight loss** | **Weight gain** |  | **Intentional weight loss** | **Unintentional weight loss** | **Weight gain** |  | **Intentional weight loss** | **Unintentional weight loss** | **Weight gain** |
| **Lipids and lipid-like molecules: Triradylcglycerols** | | | | | | | | | | | | | | | | |
| TG(56:8) | HMDB0005392 | 0.85 [0.48, 1.50] | 1.05 [0.72, 1.54] | 0.86 [0.58, 1.27] |  | 0.93 [0.52, 1.64] | 0.77 [0.53, 1.14] | 0.69 [0.47, 1.01] |  | 0.94 [0.67, 1.32] | 0.63 [0.49, 0.81] | 0.95 [0.75, 1.20] |  | 1.22 [0.81, 1.84] | 0.72 [0.54, 0.97] | 0.87 [0.67, 1.14] |
| TG(56:7) * | HMDB0005462 | 0.91 [0.53, 1.56] | 1.21 [0.83, 1.76] | 0.83 [0.57, 1.20] |  | 0.96 [0.58, 1.59] | 0.80 [0.57, 1.12] | 0.78 [0.55, 1.11] |  | 0.94 [0.67, 1.31] | 0.68 [0.53, 0.87] | 0.93 [0.74, 1.17] |  | 1.08 [0.70, 1.65] | 0.68 [0.50, 0.92] | 0.84 [0.64, 1.11] |
| TG(58:10) | HMDB0005476 | 0.97 [0.52, 1.83] | 1.21 [0.79, 1.85] | 0.98 [0.63, 1.51] |  | 0.90 [0.50, 1.62] | 0.76 [0.51, 1.14] | 0.71 [0.47, 1.07] |  | 0.87 [0.62, 1.24] | 0.67 [0.52, 0.86] | 0.98 [0.77, 1.24] |  | 1.31 [0.86, 2.01] | 0.81 [0.60, 1.08] | 0.89 [0.68, 1.17] |
| TG(58:11) | HMDB0010531 | 0.94 [0.52, 1.71] | 1.19 [0.80, 1.75] | 0.92 [0.61, 1.38] |  | 0.92 [0.52, 1.63] | 0.77 [0.52, 1.13] | 0.72 [0.49, 1.05] |  | 0.95 [0.68, 1.34] | 0.69 [0.54, 0.88] | 0.94 [0.74, 1.19] |  | 1.27 [0.84, 1.93] | 0.79 [0.59, 1.07] | 0.87 [0.66, 1.14] |
| TG(60:12) | HMDB0005478 | 0.71 [0.34, 1.48] | 1.11 [0.68, 1.79] | 0.90 [0.54, 1.50] |  | 0.80 [0.41, 1.56] | 0.76 [0.48, 1.20] | 0.71 [0.45, 1.13] |  | 0.85 [0.62, 1.17] | 0.74 [0.60, 0.93] | 0.95 [0.76, 1.18] |  | 1.39 [0.92, 2.09] | 0.85 [0.65, 1.11] | 0.86 [0.67, 1.11] |
| TG(56:10) * | HMDB0010513 | 1.04 [0.60, 1.80] | 1.22 [0.84, 1.77] | 0.88 [0.60, 1.30] |  | 0.86 [0.50, 1.49] | 0.77 [0.53, 1.10] | 0.72 [0.50, 1.03] |  | 1.00 [0.70, 1.42] | 0.70 [0.55, 0.90] | 0.93 [0.73, 1.19] |  | 1.17 [0.75, 1.83] | 0.77 [0.57, 1.04] | 0.83 [0.63, 1.11] |
| TG(58:9) | HMDB0005463 | 0.85 [0.44, 1.66] | 1.30 [0.84, 2.00] | 0.95 [0.60, 1.51] |  | 0.81 [0.43, 1.51] | 0.73 [0.47, 1.12] | 0.73 [0.47, 1.12] |  | 0.83 [0.60, 1.15] | 0.71 [0.57, 0.90] | 1.01 [0.81, 1.26] |  | 1.31 [0.88, 1.95] | 0.83 [0.63, 1.10] | 0.88 [0.67, 1.13] |
| TG(54:9) | HMDB0010498 | 1.10 [0.62, 1.93] | 1.23 [0.83, 1.81] | 0.93 [0.63, 1.36] |  | 0.77 [0.44, 1.37] | 0.77 [0.53, 1.11] | 0.70 [0.49, 1.01] |  | 1.03 [0.72, 1.48] | 0.73 [0.57, 0.93] | 0.94 [0.74, 1.21] |  | 1.00 [0.62, 1.60] | 0.77 [0.57, 1.05] | 0.83 [0.62, 1.10] |
| TG(54:6) | HMDB0005391 | 1.32 [0.76, 2.31] | 0.97 [0.68, 1.37] | 0.92 [0.65, 1.30] |  | 1.18 [0.67, 2.06] | 0.72 [0.50, 1.05] | 0.76 [0.52, 1.10] |  | 0.96 [0.68, 1.38] | 0.75 [0.58, 0.95] | 1.14 [0.89, 1.45] |  | 0.82 [0.53, 1.27] | 0.80 [0.59, 1.09] | 0.96 [0.72, 1.28] |
| TG(53:3) | HMDB0043058 | 1.80 [0.93, 3.47] | 1.22 [0.82, 1.81] | 0.82 [0.57, 1.19] |  | 1.08 [0.63, 1.84] | 0.78 [0.55, 1.11] | 0.78 [0.55, 1.11] |  | 1.29 [0.86, 1.94] | 0.79 [0.62, 1.01] | 0.92 [0.72, 1.18] |  | 0.67 [0.44, 1.00] | 0.72 [0.53, 0.98] | 0.98 [0.73, 1.32] |
| TG(54:5) | HMDB0005385 | 1.35 [0.74, 2.46] | 0.96 [0.66, 1.40] | 0.83 [0.57, 1.21] |  | 1.12 [0.64, 1.95] | 0.78 [0.53, 1.14] | 0.79 [0.54, 1.15] |  | 1.03 [0.72, 1.49] | 0.81 [0.63, 1.03] | 1.10 [0.85, 1.42] |  | 0.85 [0.54, 1.34] | 0.73 [0.54, 0.99] | 0.97 [0.73, 1.28] |
| TG(52:7) | HMDB0010517 | 1.16 [0.67, 2.01] | 1.26 [0.87, 1.83] | 0.87 [0.61, 1.25] |  | 0.91 [0.54, 1.54] | 0.79 [0.56, 1.12] | 0.75 [0.53, 1.06] |  | 1.14 [0.78, 1.67] | 0.79 [0.61, 1.03] | 0.89 [0.68, 1.16] |  | 0.82 [0.52, 1.30] | 0.78 [0.57, 1.07] | 0.84 [0.63, 1.12] |
| TG(54:2) ^¶^ | HMDB0005403 | 1.30 [0.73, 2.29] | 1.23 [0.84, 1.78] | 0.84 [0.58, 1.22] |  | 1.49 [0.91, 2.44] | 0.91 [0.67, 1.24] | 0.91 [0.66, 1.24] |  | 1.40 [0.96, 2.04] | 1.20 [0.93, 1.54] | 0.97 [0.76, 1.23] |  | 0.93 [0.59, 1.44] | 0.83 [0.62, 1.12] | 0.95 [0.72, 1.26] |
| TG(44:0) | HMDB0042063 | 1.21 [0.68, 2.15] | 1.15 [0.79, 1.67] | 0.89 [0.62, 1.29] |  | 1.07 [0.64, 1.78] | 0.83 [0.58, 1.21] | 1.25 [0.88, 1.79] |  | 1.23 [0.84, 1.80] | 1.41 [1.08, 1.85] | 0.83 [0.64, 1.08] |  | 0.95 [0.62, 1.43] | 1.21 [0.90, 1.61] | 0.95 [0.73, 1.23] |
| TG(46:0) * | HMDB0010411 | 1.09 [0.61, 1.94] | 1.10 [0.76, 1.58] | 0.94 [0.66, 1.34] |  | 1.02 [0.60, 1.72] | 0.83 [0.58, 1.19] | 1.17 [0.80, 1.73] |  | 1.27 [0.85, 1.89] | 1.51 [1.13, 2.02] | 0.89 [0.69, 1.14] |  | 0.99 [0.66, 1.48] | 1.21 [0.90, 1.62] | 0.93 [0.72, 1.19] |
| TG(46:1) ^¶^ | HMDB0010412 | 1.13 [0.65, 1.99] | 1.27 [0.87, 1.85] | 0.87 [0.60, 1.26] |  | 0.99 [0.59, 1.64] | 0.82 [0.57, 1.19] | 1.11 [0.78, 1.59] |  | 1.28 [0.87, 1.90] | 1.45 [1.10, 1.92] | 0.83 [0.64, 1.08] |  | 0.86 [0.56, 1.33] | 1.18 [0.87, 1.59] | 0.95 [0.72, 1.26] |
| **Lipids and lipid-like molecules: Glycerophosphocholines** | | | | | | | | | | | | | | | | |
| PC(38:6) | HMDB0007991 | 0.66 [0.33, 1.32] | 0.88 [0.56, 1.36] | 0.98 [0.64, 1.50] |  | 1.03 [0.61, 1.73] | 0.86 [0.60, 1.24] | 0.67 [0.47, 0.96] |  | 1.27 [0.87, 1.85] | 0.69 [0.53, 0.91] | 0.78 [0.60, 1.02] |  | 1.13 [0.77, 1.67] | 0.79 [0.61, 1.04] | 0.93 [0.72, 1.19] |
| PC(40:6) | HMDB0008057 | 0.77 [0.41, 1.45] | 0.97 [0.63, 1.48] | 0.89 [0.58, 1.35] |  | 0.95 [0.57, 1.59] | 0.85 [0.59, 1.23] | 0.70 [0.49, 1.00] |  | 1.29 [0.88, 1.90] | 0.68 [0.52, 0.89] | 0.79 [0.60, 1.02] |  | 1.11 [0.75, 1.66] | 0.84 [0.63, 1.12] | 0.86 [0.66, 1.12] |
| PC(40:9) | HMDB0008731 | 0.70 [0.36, 1.38] | 0.91 [0.59, 1.40] | 0.99 [0.65, 1.53] |  | 1.04 [0.59, 1.84] | 0.90 [0.61, 1.33] | 0.70 [0.48, 1.01] |  | 1.22 [0.84, 1.78] | 0.72 [0.57, 0.93] | 0.79 [0.62, 1.01] |  | 1.15 [0.76, 1.73] | 0.79 [0.60, 1.04] | 0.91 [0.70, 1.18] |
| PC(P-38:6)/PC(O-38:7) ^¶^ | HMDB0011229 | 0.58 [0.29, 1.16] | 0.87 [0.56, 1.36] | 1.32 [0.85, 2.05] |  | 0.60 [0.35, 1.03] | 0.71 [0.49, 1.04] | 0.70 [0.48, 1.02] |  | 0.97 [0.68, 1.38] | 0.71 [0.55, 0.92] | 0.83 [0.65, 1.07] |  | 1.23 [0.81, 1.88] | 1.15 [0.85, 1.57] | 0.96 [0.73, 1.26] |
| PC(36:0) | HMDB0008036 | 1.10 [0.65, 1.87] | 0.72 [0.47, 1.11] | 0.85 [0.58, 1.27] |  | 1.31 [0.81, 2.11] | 0.90 [0.66, 1.25] | 0.81 [0.59, 1.11] |  | 1.09 [0.74, 1.60] | 0.75 [0.57, 1.00] | 0.96 [0.73, 1.26] |  | 0.76 [0.51, 1.13] | 0.95 [0.73, 1.25] | 0.85 [0.67, 1.10] |
| LPC(24:0) | HMDB0010405 | 1.22 [0.73, 2.05] | 0.68 [0.46, 0.99] | 1.01 [0.70, 1.47] |  | 1.42 [0.86, 2.35] | 0.80 [0.58, 1.12] | 0.88 [0.63, 1.23] |  | 1.16 [0.80, 1.67] | 0.87 [0.67, 1.14] | 0.94 [0.72, 1.23] |  | 0.70 [0.46, 1.07] | 0.92 [0.69, 1.23] | 0.76 [0.59, 1.00] |
| PC(36:2) | HMDB0008039 | 1.33 [0.70, 2.53] | 0.78 [0.52, 1.17] | 0.80 [0.54, 1.19] |  | 1.35 [0.81, 2.24] | 0.78 [0.56, 1.09] | 0.65 [0.47, 0.91] |  | 1.16 [0.80, 1.67] | 0.89 [0.68, 1.16] | 0.89 [0.69, 1.17] |  | 0.66 [0.46, 0.96] | 1.00 [0.74, 1.36] | 0.86 [0.66, 1.12] |
| PC(36:4)_A | HMDB0007983 | 1.67 [0.85, 3.31] | 0.74 [0.49, 1.13] | 0.84 [0.56, 1.25] |  | 1.20 [0.74, 1.94] | 0.76 [0.56, 1.04] | 0.75 [0.55, 1.02] |  | 0.93 [0.63, 1.38] | 0.90 [0.68, 1.20] | 0.84 [0.64, 1.12] |  | 0.59 [0.39, 0.91] | 1.02 [0.75, 1.39] | 0.85 [0.65, 1.13] |
| PC(32:2) | HMDB0007874 | 1.29 [0.67, 2.50] | 1.06 [0.69, 1.63] | 0.74 [0.49, 1.11] |  | 1.18 [0.67, 2.09] | 0.73 [0.50, 1.07] | 0.65 [0.45, 0.95] |  | 1.15 [0.74, 1.79] | 1.10 [0.80, 1.51] | 0.82 [0.60, 1.12] |  | 0.57 [0.34, 0.97] | 1.06 [0.74, 1.51] | 0.94 [0.68, 1.31] |
| LPC(14:0) | HMDB0010379 | 1.16 [0.65, 2.06] | 1.15 [0.77, 1.72] | 0.74 [0.52, 1.08] |  | 1.40 [0.86, 2.29] | 0.73 [0.51, 1.04] | 0.89 [0.63, 1.27] |  | 1.15 [0.76, 1.75] | 1.08 [0.80, 1.45] | 0.81 [0.61, 1.08] |  | 0.70 [0.44, 1.12] | 1.19 [0.86, 1.64] | 0.82 [0.61, 1.10] |
| PC(36:1) | HMDB0008038 | 1.26 [0.71, 2.26] | 1.11 [0.75, 1.65] | 0.89 [0.60, 1.32] |  | 1.32 [0.83, 2.08] | 0.92 [0.66, 1.29] | 0.80 [0.58, 1.12] |  | 1.18 [0.80, 1.74] | 1.04 [0.79, 1.38] | 0.79 [0.60, 1.03] |  | 0.72 [0.48, 1.10] | 1.05 [0.78, 1.41] | 0.85 [0.65, 1.10] |
| PC(30:1) | HMDB0007870 | 1.19 [0.64, 2.19] | 1.37 [0.89, 2.10] | 0.85 [0.57, 1.26] |  | 0.95 [0.56, 1.60] | 0.89 [0.61, 1.30] | 0.86 [0.59, 1.25] |  | 1.14 [0.74, 1.75] | 1.33 [0.98, 1.81] | 0.76 [0.57, 1.02] |  | 0.73 [0.45, 1.19] | 1.22 [0.88, 1.71] | 0.94 [0.70, 1.27] |
| LPC(22:5) | HMDB0010402 | 1.64 [0.77, 3.48] | 1.14 [0.77, 1.70] | 1.38 [0.88, 2.15] |  | 1.00 [0.60, 1.66] | 1.26 [0.84, 1.90] | 1.03 [0.70, 1.52] |  | 1.00 [0.69, 1.44] | 1.54 [1.15, 2.06] | 0.97 [0.76, 1.23] |  | 0.68 [0.49, 0.96] | 1.10 [0.82, 1.48] | 1.02 [0.78, 1.33] |
| **Lipids and lipid-like molecules: Glycerophosphoethanolamines** | | | | | | | | | | | | | | | | |
| PE(P-38:6)/PE(O-38:7) | HMDB0011420 | 0.89 [0.45, 1.77] | 0.95 [0.63, 1.43] | 1.06 [0.70, 1.61] |  | 0.85 [0.49, 1.48] | 0.66 [0.45, 0.98] | 0.57 [0.38, 0.84] |  | 1.12 [0.76, 1.66] | 0.69 [0.52, 0.93] | 0.83 [0.63, 1.10] |  | 1.27 [0.83, 1.94] | 1.00 [0.75, 1.35] | 0.96 [0.73, 1.26] |
| PE(38:2) | HMDB0008942 | 1.70 [0.92, 3.13] | 0.97 [0.65, 1.47] | 1.04 [0.70, 1.54] |  | 1.31 [0.80, 2.16] | 0.77 [0.56, 1.06] | 0.69 [0.50, 0.94] |  | 1.32 [0.90, 1.92] | 0.79 [0.60, 1.04] | 0.91 [0.70, 1.19] |  | 0.66 [0.44, 0.98] | 0.92 [0.68, 1.25] | 0.93 [0.70, 1.22] |
| LPE(20:4) | HMDB0011517 | 1.86 [0.94, 3.66] | 1.62 [1.02, 2.56] | 1.46 [0.94, 2.25] |  | 1.30 [0.84, 2.00] | 1.20 [0.88, 1.65] | 0.99 [0.73, 1.36] |  | 1.23 [0.85, 1.79] | 1.24 [0.94, 1.62] | 1.02 [0.78, 1.33] |  | 0.80 [0.54, 1.19] | 1.06 [0.80, 1.40] | 0.85 [0.66, 1.09] |
| **Lipids and lipid-like molecules: Glycerophosphoserines** | | | | | | | | | | | | | | | | |
| PS(34:0) * | HMDB0012356 | 0.73 [0.40, 1.33] | 1.22 [0.84, 1.77] | 0.93 [0.63, 1.38] |  | 1.16 [0.71, 1.88] | 0.89 [0.63, 1.25] | 0.75 [0.54, 1.05] |  | 1.10 [0.77, 1.58] | 0.78 [0.61, 1.00] | 0.87 [0.68, 1.11] |  | 0.95 [0.60, 1.50] | 0.72 [0.53, 0.98] | 0.83 [0.62, 1.10] |
| **Lipids and lipid-like molecules: Phosphosphingolipids** | | | | | | | | | | | | | | | | |
| SM(d18:1/24:0) | HMDB0011697 | 0.94 [0.53, 1.68] | 0.63 [0.43, 0.92] | 0.85 [0.59, 1.23] |  | 1.14 [0.67, 1.95] | 0.66 [0.47, 0.94] | 0.66 [0.47, 0.93] |  | 1.27 [0.89, 1.81] | 0.91 [0.71, 1.17] | 1.01 [0.79, 1.29] |  | 0.78 [0.52, 1.18] | 0.91 [0.67, 1.25] | 0.85 [0.64, 1.12] |
| SM(d18:1/22:0) | HMDB0012103 | 0.99 [0.54, 1.81] | 0.60 [0.40, 0.88] | 0.95 [0.65, 1.38] |  | 1.29 [0.75, 2.24] | 0.77 [0.54, 1.09] | 0.64 [0.46, 0.91] |  | 1.20 [0.83, 1.73] | 0.92 [0.71, 1.19] | 1.02 [0.79, 1.33] |  | 0.79 [0.52, 1.18] | 0.92 [0.68, 1.25] | 0.92 [0.70, 1.21] |
| SM(d18:1/20:0) | HMDB0012102 | 1.00 [0.56, 1.79] | 0.66 [0.45, 0.96] | 1.06 [0.73, 1.56] |  | 1.30 [0.76, 2.22] | 0.85 [0.61, 1.20] | 0.72 [0.52, 1.00] |  | 1.20 [0.82, 1.77] | 0.88 [0.68, 1.16] | 1.02 [0.78, 1.33] |  | 0.78 [0.52, 1.15] | 0.90 [0.67, 1.21] | 0.92 [0.70, 1.20] |
| SM(d18:1/16:1) ^¶^ | HMDB0240613 | 1.04 [0.54, 1.97] | 0.55 [0.36, 0.85] | 1.02 [0.67, 1.56] |  | 1.25 [0.74, 2.13] | 1.03 [0.71, 1.50] | 0.66 [0.46, 0.94] |  | 1.06 [0.70, 1.60] | 0.78 [0.58, 1.05] | 0.97 [0.72, 1.30] |  | 0.73 [0.49, 1.08] | 0.98 [0.72, 1.33] | 0.98 [0.75, 1.30] |
| SM(d18:1/22:1) | HMDB0012104 | 1.11 [0.60, 2.05] | 0.59 [0.39, 0.88] | 1.11 [0.74, 1.66] |  | 1.21 [0.72, 2.04] | 0.95 [0.67, 1.36] | 0.69 [0.49, 0.97] |  | 1.12 [0.75, 1.67] | 0.83 [0.62, 1.11] | 1.04 [0.78, 1.38] |  | 0.77 [0.52, 1.15] | 0.95 [0.70, 1.29] | 0.97 [0.74, 1.27] |
| **Lipids and lipid-like molecules: Steroid esters** | | | | | | | | | | | | | | | | |
| CE(20:5) | HMDB0006731 | 0.56 [0.30, 1.05] | 0.81 [0.53, 1.24] | 0.93 [0.62, 1.41] |  | 0.83 [0.47, 1.45] | 0.92 [0.61, 1.40] | 0.88 [0.58, 1.34] |  | 0.97 [0.70, 1.34] | 0.79 [0.63, 1.01] | 1.00 [0.79, 1.27] |  | 1.32 [0.86, 2.03] | 0.91 [0.69, 1.19] | 0.80 [0.62, 1.03] |
| CE(18:0) ^¶^ | HMDB0010368 | 0.73 [0.40, 1.31] | 0.50 [0.32, 0.76] | 0.91 [0.60, 1.38] |  | 0.99 [0.62, 1.60] | 0.99 [0.69, 1.40] | 0.73 [0.52, 1.00] |  | 0.95 [0.66, 1.37] | 0.82 [0.64, 1.06] | 1.12 [0.86, 1.46] |  | 0.64 [0.44, 0.93] | 1.04 [0.78, 1.40] | 0.89 [0.69, 1.16] |
| CE(22:6) ^¶^ | HMDB0006733 | 0.54 [0.25, 1.17] | 0.54 [0.33, 0.89] | 1.14 [0.67, 1.94] |  | 0.58 [0.31, 1.10] | 0.88 [0.55, 1.40] | 0.78 [0.49, 1.22] |  | 0.91 [0.64, 1.28] | 0.77 [0.60, 0.98] | 1.01 [0.78, 1.30] |  | 1.38 [0.89, 2.14] | 1.07 [0.80, 1.42] | 0.87 [0.67, 1.12] |
| CE(22:4) | HMDB0006729 | 1.15 [0.55, 2.39] | 0.79 [0.51, 1.21] | 1.17 [0.74, 1.86] |  | 0.94 [0.48, 1.84] | 0.84 [0.51, 1.37] | 1.21 [0.73, 2.01] |  | 0.81 [0.63, 1.04] | 0.92 [0.73, 1.16] | 1.19 [0.89, 1.59] |  | 0.71 [0.51, 0.99] | 1.11 [0.80, 1.54] | 0.91 [0.70, 1.20] |
| CE(20:5) | HMDB0006731 | 0.56 [0.30, 1.05] | 0.81 [0.53, 1.24] | 0.93 [0.62, 1.41] |  | 0.83 [0.47, 1.45] | 0.92 [0.61, 1.40] | 0.88 [0.58, 1.34] |  | 0.97 [0.70, 1.34] | 0.79 [0.63, 1.01] | 1.00 [0.79, 1.27] |  | 1.32 [0.86, 2.03] | 0.91 [0.69, 1.19] | 0.80 [0.62, 1.03] |
| **Lipids and lipid-like molecules: Fatty acid esters** | | | | | | | | | | | | | | | | |
| CAR(14:2) * | HMDB0013331 | 0.37 [0.19, 0.73] | 1.13 [0.77, 1.67] | 1.29 [0.88, 1.90] |  | 1.01 [0.63, 1.63] | 1.34 [0.96, 1.87] | 0.78 [0.56, 1.08] |  | 0.74 [0.52, 1.06] | 0.78 [0.60, 1.02] | 1.08 [0.83, 1.41] |  | 0.92 [0.62, 1.37] | 0.96 [0.72, 1.27] | 1.22 [0.94, 1.59] |
| CAR(6:0) | HMDB0000705 | 0.77 [0.42, 1.41] | 1.47 [1.03, 2.09] | 1.38 [0.98, 1.94] |  | 0.97 [0.61, 1.57] | 1.33 [0.99, 1.77] | 0.81 [0.58, 1.13] |  | 0.96 [0.65, 1.42] | 1.01 [0.76, 1.34] | 1.01 [0.76, 1.34] |  | 1.01 [0.65, 1.55] | 1.11 [0.82, 1.50] | 1.27 [0.98, 1.66] |
| CAR(4:0(OH)) | HMDB0013127 | 0.80 [0.46, 1.37] | 1.21 [0.85, 1.72] | 1.41 [0.98, 2.03] |  | 1.23 [0.76, 1.99] | 1.63 [1.16, 2.28] | 1.03 [0.75, 1.41] |  | 0.74 [0.49, 1.12] | 1.16 [0.88, 1.53] | 1.09 [0.83, 1.43] |  | 1.10 [0.70, 1.72] | 1.01 [0.76, 1.35] | 1.24 [0.94, 1.64] |
| **Lipids and lipid-like molecules: Fatty acids and conjugates** | | | | | | | | | | | | | | | | |
| Azelaic acid | HMDB0000784 | 1.21 [0.69, 2.11] | 0.99 [0.72, 1.35] | 0.74 [0.57, 0.96] |  | 1.48 [0.88, 2.50] | 1.35 [0.95, 1.92] | 0.91 [0.69, 1.19] |  | 0.70 [0.50, 0.97] | 0.98 [0.74, 1.29] | 0.97 [0.74, 1.28] |  | 0.71 [0.49, 1.02] | 1.14 [0.80, 1.62] | 0.82 [0.64, 1.06] |
| Adipic acid or Methylglutaric acid | HMDB0000448 | 0.92 [0.47, 1.82] | 0.82 [0.53, 1.26] | 1.23 [0.84, 1.79] |  | 1.32 [0.81, 2.14] | 1.60 [1.11, 2.30] | 1.00 [0.67, 1.49] |  | 1.03 [0.65, 1.62] | 1.16 [0.85, 1.58] | 1.46 [1.08, 1.98] |  | 0.96 [0.65, 1.43] | 1.09 [0.81, 1.48] | 1.15 [0.89, 1.49] |
| 3-Methyladipic acid or Pimelic acid | HMDB0000555 | 1.42 [0.83, 2.43] | 1.27 [0.90, 1.79] | 0.97 [0.65, 1.46] |  | 1.05 [0.63, 1.75] | 1.17 [0.80, 1.70] | 0.62 [0.41, 0.92] |  | 1.00 [0.67, 1.47] | 1.25 [0.94, 1.65] | 1.00 [0.75, 1.33] |  | 0.76 [0.48, 1.19] | 1.22 [0.91, 1.62] | 1.03 [0.78, 1.35] |
| Suberic acid | HMDB0000893 | 1.21 [0.65, 2.26] | 1.24 [0.85, 1.80] | 1.14 [0.78, 1.66] |  | 0.96 [0.55, 1.69] | 1.59 [1.07, 2.35] | 1.07 [0.73, 1.58] |  | 0.55 [0.34, 0.90] | 0.95 [0.71, 1.27] | 1.11 [0.86, 1.44] |  | 0.70 [0.43, 1.15] | 1.26 [0.92, 1.72] | 1.11 [0.83, 1.48] |
| Juniperic acid | HMDB0006294 | 0.75 [0.40, 1.41] | 1.07 [0.72, 1.58] | 1.40 [0.96, 2.03] |  | 1.15 [0.68, 1.95] | 1.98 [1.35, 2.91] | 1.34 [0.91, 1.96] |  | 0.87 [0.64, 1.18] | 1.12 [0.88, 1.42] | 1.00 [0.81, 1.24] |  | 1.22 [0.80, 1.87] | 1.14 [0.84, 1.54] | 1.24 [0.94, 1.64] |
| Lauric acid | HMDB0000638 | 0.93 [0.50, 1.72] | 1.25 [0.83, 1.89] | 1.40 [0.92, 2.14] |  | 1.09 [0.66, 1.82] | 1.21 [0.87, 1.67] | 1.19 [0.86, 1.64] |  | 0.91 [0.60, 1.36] | 1.25 [0.95, 1.65] | 0.96 [0.72, 1.28] |  | 0.96 [0.63, 1.47] | 1.23 [0.92, 1.65] | 1.19 [0.91, 1.55] |
| Tetradecanedioic acid* | HMDB0000872 | 0.80 [0.36, 1.77] | 1.17 [0.79, 1.72] | 1.36 [0.95, 1.96] |  | 1.24 [0.69, 2.25] | 2.31 [1.55, 3.45] | 1.44 [0.95, 2.20] |  | 0.79 [0.53, 1.19] | 1.02 [0.80, 1.30] | 1.03 [0.81, 1.31] |  | 1.15 [0.80, 1.66] | 1.12 [0.84, 1.49] | 1.15 [0.89, 1.49] |
| Hexadecanedioic acid* | HMDB0000672 | 0.88 [0.46, 1.68] | 1.10 [0.74, 1.62] | 1.22 [0.84, 1.76] |  | 1.28 [0.76, 2.14] | 2.18 [1.50, 3.17] | 1.44 [0.98, 2.11] |  | 0.81 [0.55, 1.21] | 1.00 [0.78, 1.29] | 1.07 [0.84, 1.36] |  | 1.22 [0.86, 1.74] | 1.17 [0.88, 1.55] | 1.24 [0.96, 1.59] |
| Sebacic acid^¶^ | HMDB0000792 | 1.17 [0.70, 1.96] | 1.11 [0.79, 1.56] | 0.84 [0.61, 1.16] |  | 1.38 [0.85, 2.24] | 2.05 [1.43, 2.95] | 1.16 [0.82, 1.63] |  | 0.71 [0.49, 1.04] | 0.98 [0.75, 1.28] | 1.12 [0.86, 1.45] |  | 0.75 [0.48, 1.16] | 1.47 [1.08, 2.01] | 1.08 [0.82, 1.44] |
| **Lipids and lipid-like molecules: Bile acids, alcohols and derivatives** | | | | | | | | | | | | | | | | |
| Hyodeoxycholic acid/Ursodeoxycholic acid | HMDB0000733 | 1.78 [1.03, 3.07] | 0.97 [0.66, 1.43] | 1.00 [0.69, 1.44] |  | 1.46 [0.89, 2.40] | 1.08 [0.77, 1.54] | 1.04 [0.73, 1.47] |  | 1.41 [0.96, 2.09] | 1.22 [0.93, 1.60] | 1.20 [0.91, 1.58] |  | 1.06 [0.70, 1.59] | 0.91 [0.69, 1.20] | 1.09 [0.84, 1.41] |
| **Organic acids and derivatives: Amino acids, peptides, and analogues** | | | | | | | | | | | | | | | | |
| Tryptophan | HMDB0000929 | 0.52 [0.30, 0.91] | 0.80 [0.55, 1.16] | 0.89 [0.61, 1.32] |  | 1.13 [0.65, 1.94] | 0.74 [0.53, 1.04] | 0.91 [0.63, 1.30] |  | 0.79 [0.56, 1.13] | 0.82 [0.62, 1.09] | 1.41 [1.03, 1.94] |  | 1.01 [0.62, 1.64] | 0.85 [0.62, 1.18] | 0.75 [0.56, 1.01] |
| Valine | HMDB0000883 | 0.62 [0.35, 1.08] | 1.12 [0.73, 1.72] | 0.84 [0.55, 1.27] |  | 1.42 [0.80, 2.53] | 0.64 [0.45, 0.91] | 0.66 [0.46, 0.94] |  | 0.90 [0.64, 1.26] | 0.92 [0.71, 1.20] | 1.00 [0.76, 1.31] |  | 0.98 [0.65, 1.49] | 0.86 [0.66, 1.14] | 0.85 [0.66, 1.09] |
| Homoarginine | HMDB0000670 | 0.64 [0.39, 1.05] | 0.78 [0.53, 1.17] | 0.73 [0.49, 1.07] |  | 1.27 [0.75, 2.15] | 1.01 [0.73, 1.40] | 0.80 [0.59, 1.07] |  | 0.85 [0.59, 1.23] | 0.89 [0.67, 1.19] | 1.02 [0.76, 1.37] |  | 0.79 [0.53, 1.16] | 0.72 [0.54, 0.96] | 0.91 [0.69, 1.20] |
| Asparagine | HMDB0000168 | 0.66 [0.42, 1.04] | 1.00 [0.68, 1.48] | 0.89 [0.61, 1.30] |  | 0.95 [0.59, 1.51] | 0.85 [0.61, 1.19] | 0.70 [0.50, 0.96] |  | 0.91 [0.67, 1.24] | 0.98 [0.75, 1.27] | 1.04 [0.79, 1.37] |  | 1.03 [0.69, 1.55] | 0.74 [0.56, 0.98] | 0.80 [0.62, 1.05] |
| Dimethylglycine | HMDB0000092 | 0.85 [0.49, 1.48] | 1.21 [0.81, 1.81] | 1.45 [1.00, 2.11] |  | 1.26 [0.84, 1.87] | 1.14 [0.83, 1.55] | 1.09 [0.80, 1.49] |  | 0.83 [0.57, 1.21] | 1.19 [0.90, 1.56] | 1.13 [0.86, 1.47] |  | 1.40 [0.95, 2.07] | 1.11 [0.82, 1.49] | 1.13 [0.86, 1.49] |
| N-Formylmethionine | HMDB0001015 | 1.54 [0.81, 2.94] | 1.66 [1.08, 2.56] | 1.07 [0.70, 1.62] |  | 1.12 [0.70, 1.79] | 1.01 [0.70, 1.44] | 1.31 [0.91, 1.87] |  | 0.87 [0.56, 1.37] | 1.42 [1.03, 1.96] | 0.82 [0.63, 1.06] |  | 1.89 [1.12, 3.19] | 1.21 [0.84, 1.75] | 1.36 [0.97, 1.92] |
| Ornithine | HMDB0000214 | 0.97 [0.58, 1.63] | 1.42 [0.95, 2.12] | 0.83 [0.61, 1.14] |  | 1.39 [0.85, 2.28] | 1.19 [0.87, 1.63] | 1.07 [0.80, 1.43] |  | 0.85 [0.63, 1.15] | 1.05 [0.79, 1.40] | 1.09 [0.82, 1.46] |  | 0.89 [0.60, 1.33] | 1.21 [0.90, 1.64] | 0.78 [0.61, 1.01] |
| **Organic acids and derivatives: Other metabolites** | | | | | | | | | | | | | | | | |
| Levulinic acid | HMDB0000720 | 0.59 [0.32, 1.10] | 0.93 [0.61, 1.40] | 0.88 [0.58, 1.33] |  | 1.37 [0.78, 2.40] | 0.75 [0.51, 1.12] | 0.65 [0.44, 0.97] |  | 1.07 [0.70, 1.62] | 0.97 [0.78, 1.20] | 0.95 [0.77, 1.17] |  | 1.33 [0.81, 2.18] | 0.93 [0.66, 1.31] | 0.82 [0.60, 1.13] |
| N-Acetylcarnosine^¶^ | HMDB0012881 | 1.63 [0.68, 3.93] | 0.67 [0.41, 1.11] | 0.97 [0.58, 1.61] |  | 1.09 [0.65, 1.82] | 1.17 [0.80, 1.72] | 0.80 [0.59, 1.08] |  | 0.72 [0.44, 1.18] | 0.76 [0.53, 1.10] | 0.58 [0.41, 0.81] |  | 1.20 [0.71, 2.05] | 1.07 [0.77, 1.50] | 0.96 [0.71, 1.28] |
| Ketoisovaleric acid* | HMDB0000019 | 0.91 [0.53, 1.54] | 0.71 [0.48, 1.06] | 1.19 [0.81, 1.73] |  | 0.78 [0.46, 1.34] | 0.86 [0.59, 1.25] | 0.57 [0.39, 0.84] |  | 0.90 [0.62, 1.31] | 1.25 [0.98, 1.59] | 0.73 [0.55, 0.95] |  | 0.83 [0.54, 1.27] | 0.99 [0.73, 1.33] | 0.94 [0.72, 1.24] |
| 2-Hydroxyglutaric acid | HMDB0000694 | 1.61 [0.87, 2.99] | 1.24 [0.81, 1.90] | 1.18 [0.78, 1.78] |  | 1.16 [0.70, 1.91] | 1.04 [0.73, 1.49] | 0.75 [0.52, 1.09] |  | 0.84 [0.58, 1.22] | 1.23 [0.95, 1.61] | 1.07 [0.82, 1.38] |  | 0.79 [0.50, 1.23] | 1.20 [0.90, 1.61] | 0.94 [0.71, 1.24] |
| cis-Aconitic acid | HMDB0000072 | 1.30 [0.70, 2.44] | 1.17 [0.76, 1.79] | 1.11 [0.74, 1.67] |  | 0.92 [0.57, 1.48] | 1.28 [0.90, 1.83] | 0.87 [0.62, 1.23] |  | 0.82 [0.55, 1.22] | 1.12 [0.85, 1.47] | 1.16 [0.89, 1.52] |  | 0.86 [0.54, 1.38] | 1.21 [0.88, 1.66] | 1.26 [0.94, 1.69] |
| 3S-Hydroxyhexanoic acid* | HMDB0010718 | 1.23 [0.66, 2.29] | 1.21 [0.81, 1.82] | 0.90 [0.61, 1.34] |  | 1.16 [0.71, 1.90] | 2.13 [1.49, 3.04] | 1.26 [0.88, 1.79] |  | 0.74 [0.51, 1.09] | 0.97 [0.73, 1.28] | 1.20 [0.91, 1.58] |  | 1.06 [0.69, 1.64] | 1.03 [0.76, 1.40] | 1.31 [0.98, 1.74] |
| 3-Hydroxyoctanoic acid* | HMDB0001954 | 1.00 [0.58, 1.72] | 1.15 [0.77, 1.73] | 0.85 [0.58, 1.24] |  | 1.14 [0.70, 1.86] | 1.94 [1.34, 2.79] | 1.08 [0.76, 1.55] |  | 0.82 [0.60, 1.14] | 1.05 [0.80, 1.37] | 1.28 [0.97, 1.69] |  | 1.05 [0.68, 1.61] | 1.07 [0.79, 1.44] | 1.21 [0.92, 1.59] |
| N1-Acetylspermidine | HMDB0001276 | 0.45 [0.23, 0.89] | 1.36 [0.88, 2.08] | 1.11 [0.72, 1.71] |  | 1.47 [0.88, 2.44] | 1.21 [0.85, 1.72] | 1.38 [0.97, 1.96] |  | 0.91 [0.65, 1.27] | 1.25 [0.96, 1.62] | 1.22 [0.94, 1.57] |  | 1.27 [0.79, 2.02] | 1.21 [0.89, 1.66] | 1.34 [0.99, 1.80] |
| Fumaric acid or Maleic acid | HMDB0000134 | 1.35 [0.78, 2.34] | 1.33 [0.91, 1.95] | 1.37 [0.94, 2.00] |  | 0.80 [0.51, 1.27] | 1.02 [0.74, 1.39] | 0.80 [0.57, 1.12] |  | 1.09 [0.74, 1.59] | 1.28 [0.99, 1.66] | 0.92 [0.71, 1.21] |  | 0.91 [0.58, 1.41] | 1.31 [0.95, 1.81] | 1.28 [0.96, 1.71] |
| Malic acid | HMDB0000156 | 1.35 [0.78, 2.35] | 1.36 [0.93, 1.99] | 1.27 [0.86, 1.86] |  | 0.74 [0.47, 1.17] | 1.03 [0.75, 1.41] | 0.82 [0.59, 1.14] |  | 1.03 [0.70, 1.52] | 1.34 [1.04, 1.73] | 0.96 [0.73, 1.25] |  | 0.98 [0.63, 1.53] | 1.30 [0.94, 1.80] | 1.32 [0.98, 1.77] |
| **Nucleosides, nucleotides, and analogues** | | | | | | | | | | | | | | | | |
| Uridine | HMDB0000296 | 0.78 [0.50, 1.21] | 0.72 [0.52, 0.99] | 0.91 [0.65, 1.28] |  | 0.97 [0.61, 1.56] | 0.71 [0.52, 0.97] | 0.94 [0.68, 1.30] |  | 1.01 [0.68, 1.49] | 0.78 [0.60, 1.01] | 1.02 [0.77, 1.34] |  | 0.76 [0.50, 1.15] | 0.76 [0.57, 1.01] | 0.75 [0.58, 0.98] |
| ADP | HMDB0001341 | 0.90 [0.53, 1.52] | 1.00 [0.63, 1.57] | 1.75 [0.95, 3.19] |  | 0.74 [0.47, 1.15] | 0.84 [0.59, 1.21] | 0.85 [0.60, 1.20] |  | 1.07 [0.72, 1.59] | 0.85 [0.67, 1.07] | 0.85 [0.68, 1.07] |  | 1.50 [0.81, 2.75] | 0.78 [0.61, 0.99] | 1.02 [0.78, 1.33] |
| ATP | HMDB0000538 | 0.90 [0.60, 1.36] | 0.97 [0.67, 1.40] | 1.96 [0.99, 3.87] |  | 0.76 [0.49, 1.18] | 0.94 [0.65, 1.36] | 0.85 [0.61, 1.19] |  | 0.96 [0.68, 1.36] | 0.85 [0.68, 1.06] | 0.89 [0.71, 1.12] |  | 1.51 [0.74, 3.09] | 0.73 [0.56, 0.95] | 1.02 [0.74, 1.39] |
| N4-Acetylcytidine | HMDB0005923 | 0.71 [0.38, 1.35] | 1.29 [0.86, 1.92] | 1.22 [0.82, 1.79] |  | 1.14 [0.71, 1.84] | 1.49 [1.06, 2.09] | 1.01 [0.72, 1.41] |  | 0.73 [0.49, 1.09] | 1.20 [0.90, 1.60] | 1.23 [0.92, 1.64] |  | 1.02 [0.67, 1.55] | 1.01 [0.75, 1.35] | 1.08 [0.83, 1.42] |
| Pseudouridine | HMDB0000767 | 0.99 [0.53, 1.84] | 1.25 [0.83, 1.89] | 1.29 [0.86, 1.94] |  | 1.49 [0.91, 2.44] | 1.28 [0.88, 1.86] | 0.99 [0.68, 1.43] |  | 0.75 [0.49, 1.15] | 1.27 [0.95, 1.71] | 1.06 [0.79, 1.43] |  | 0.94 [0.56, 1.57] | 1.33 [0.94, 1.87] | 1.05 [0.76, 1.45] |
| **Benzenoids** | | | | | | | | | | | | | | | | |
| Homovanillic acid | HMDB0000118 | 0.85 [0.46, 1.57] | 1.34 [0.87, 2.08] | 0.80 [0.52, 1.22] |  | 0.90 [0.54, 1.49] | 1.23 [0.86, 1.77] | 0.98 [0.68, 1.40] |  | 0.88 [0.57, 1.34] | 1.02 [0.76, 1.36] | 1.02 [0.76, 1.36] |  | 0.80 [0.51, 1.25] | 1.42 [1.03, 1.94] | 1.31 [0.98, 1.76] |
| **Organic oxygen compounds: Carbohydrates and carbohydrate conjugates** | | | | | | | | | | | | | | | | |
| Myo-inositol | HMDB0000211 | 0.97 [0.54, 1.77] | 1.27 [0.90, 1.80] | 0.83 [0.53, 1.30] |  | 1.19 [0.83, 1.71] | 1.02 [0.73, 1.43] | 1.07 [0.77, 1.47] |  | 0.84 [0.54, 1.31] | 1.11 [0.84, 1.45] | 1.00 [0.75, 1.33] |  | 0.75 [0.44, 1.28] | 1.25 [0.96, 1.62] | 1.22 [0.95, 1.57] |
| Glyceric acid | HMDB0000139 | 0.93 [0.56, 1.54] | 0.67 [0.46, 0.99] | 1.01 [0.73, 1.41] |  | 1.63 [1.05, 2.55] | 0.75 [0.52, 1.08] | 1.22 [0.88, 1.69] |  | 1.06 [0.71, 1.59] | 0.96 [0.73, 1.27] | 1.14 [0.88, 1.49] |  | 1.03 [0.68, 1.55] | 0.90 [0.67, 1.22] | 0.57 [0.41, 0.78] |
| Hexose | HMDB0000122 | 1.10 [0.68, 1.77] | 1.11 [0.76, 1.62] | 0.91 [0.63, 1.31] |  | 1.03 [0.70, 1.51] | 0.98 [0.73, 1.32] | 1.05 [0.80, 1.39] |  | 0.99 [0.66, 1.48] | 1.31 [1.00, 1.72] | 0.97 [0.72, 1.30] |  | 0.55 [0.33, 0.91] | 1.24 [0.91, 1.68] | 1.13 [0.85, 1.52] |
| Adonitol or Arabitol^¶^ | HMDB0000508 | 0.80 [0.42, 1.52] | 1.52 [1.03, 2.25] | 0.89 [0.58, 1.38] |  | 1.35 [0.83, 2.21] | 0.85 [0.58, 1.26] | 0.94 [0.65, 1.36] |  | 0.87 [0.57, 1.33] | 1.34 [1.02, 1.75] | 1.12 [0.85, 1.48] |  | 0.91 [0.55, 1.52] | 1.12 [0.80, 1.57] | 0.96 [0.70, 1.31] |
| Glucuronic acid | HMDB0000127 | 1.23 [0.71, 2.16] | 1.33 [0.97, 1.82] | 0.86 [0.57, 1.29] |  | 1.32 [0.92, 1.90] | 0.99 [0.72, 1.36] | 0.70 [0.49, 1.01] |  | 1.19 [0.82, 1.72] | 1.17 [0.88, 1.56] | 1.02 [0.76, 1.38] |  | 1.10 [0.70, 1.72] | 1.25 [0.92, 1.70] | 1.36 [1.04, 1.78] |
| Sorbitol | HMDB0000247 | 0.96 [0.44, 2.08] | 1.23 [0.82, 1.83] | 0.75 [0.44, 1.31] |  | 0.74 [0.42, 1.31] | 0.77 [0.53, 1.14] | 0.80 [0.55, 1.16] |  | 1.06 [0.76, 1.48] | 1.37 [1.11, 1.69] | 1.09 [0.86, 1.39] |  | 1.12 [0.78, 1.60] | 1.23 [0.95, 1.59] | 1.22 [0.97, 1.54] |
| Threitol | HMDB0004136 | 1.25 [0.69, 2.27] | 1.66 [1.12, 2.46] | 1.10 [0.74, 1.64] |  | 0.98 [0.60, 1.59] | 0.93 [0.66, 1.33] | 1.03 [0.73, 1.44] |  | 0.95 [0.61, 1.48] | 1.47 [1.10, 1.96] | 1.18 [0.88, 1.58] |  | 0.94 [0.60, 1.47] | 1.24 [0.91, 1.69] | 1.00 [0.74, 1.34] |
| Sucrose or Lactose or Trehalose | HMDB0000258 | 1.37 [0.85, 2.22] | 1.40 [1.00, 1.98] | 1.09 [0.76, 1.57] |  | 1.19 [0.78, 1.82] | 1.00 [0.72, 1.37] | 1.08 [0.79, 1.47] |  | 0.89 [0.59, 1.35] | 1.38 [1.06, 1.79] | 1.06 [0.80, 1.40] |  | 1.08 [0.69, 1.69] | 1.29 [0.96, 1.73] | 1.08 [0.81, 1.45] |
| **Organoheterocyclic compounds** | | | | | | | | | | | | | | | | |
| Uracil | HMDB0000300 | 0.73 [0.47, 1.15] | 0.74 [0.53, 1.02] | 0.91 [0.65, 1.28] |  | 0.96 [0.60, 1.55] | 0.74 [0.54, 1.02] | 0.92 [0.66, 1.28] |  | 0.99 [0.66, 1.46] | 0.77 [0.59, 1.01] | 1.02 [0.77, 1.35] |  | 0.72 [0.47, 1.10] | 0.76 [0.56, 1.01] | 0.69 [0.52, 0.90] |
| Niacinamide | HMDB0001406 | 0.65 [0.34, 1.24] | 0.72 [0.47, 1.09] | 1.06 [0.72, 1.57] |  | 1.17 [0.72, 1.88] | 0.87 [0.63, 1.20] | 0.89 [0.65, 1.22] |  | 0.93 [0.65, 1.31] | 0.87 [0.68, 1.11] | 0.79 [0.62, 1.01] |  | 1.12 [0.74, 1.71] | 0.66 [0.48, 0.91] | 1.25 [0.95, 1.64] |
| Serotonin | HMDB0000259 | 0.85 [0.53, 1.36] | 0.89 [0.61, 1.30] | 1.03 [0.67, 1.57] |  | 0.76 [0.46, 1.25] | 0.85 [0.57, 1.28] | 0.90 [0.60, 1.36] |  | 0.88 [0.64, 1.20] | 0.90 [0.70, 1.15] | 0.91 [0.71, 1.17] |  | 1.08 [0.66, 1.78] | 0.74 [0.57, 0.96] | 0.78 [0.61, 1.00] |
| 1-Methyl nicotinamide^¶^ | HMDB0000699 | 0.51 [0.26, 1.01] | 0.79 [0.52, 1.20] | 1.04 [0.70, 1.56] |  | 1.12 [0.67, 1.87] | 0.83 [0.57, 1.21] | 0.99 [0.70, 1.42] |  | 1.14 [0.84, 1.56] | 1.03 [0.83, 1.29] | 0.84 [0.66, 1.08] |  | 0.81 [0.51, 1.30] | 0.57 [0.39, 0.82] | 1.04 [0.78, 1.38] |
| Acisoga | HMDB0061384 | 0.52 [0.28, 0.97] | 1.77 [1.13, 2.78] | 1.57 [1.03, 2.41] |  | 1.89 [1.14, 3.15] | 1.25 [0.89, 1.75] | 1.38 [0.98, 1.95] |  | 1.00 [0.70, 1.42] | 0.90 [0.70, 1.16] | 1.07 [0.82, 1.38] |  | 1.03 [0.66, 1.61] | 1.13 [0.83, 1.54] | 1.17 [0.88, 1.56] |
| Theophylline | HMDB0001889 | 1.18 [0.70, 2.01] | 1.31 [0.92, 1.86] | 0.67 [0.44, 1.02] |  | 1.27 [0.85, 1.90] | 1.16 [0.84, 1.59] | 1.33 [0.98, 1.80] |  | 0.99 [0.66, 1.50] | 1.02 [0.78, 1.33] | 0.96 [0.73, 1.25] |  | 0.84 [0.54, 1.33] | 1.25 [0.93, 1.67] | 1.24 [0.95, 1.63] |
| Glucosan or 3-Hydroxymethylglutaric acid | HMDB0000640 | 1.19 [0.60, 2.33] | 1.84 [1.19, 2.84] | 1.36 [0.88, 2.12] |  | 1.20 [0.69, 2.09] | 1.12 [0.74, 1.69] | 0.90 [0.59, 1.37] |  | 0.77 [0.48, 1.24] | 1.21 [0.92, 1.58] | 1.08 [0.82, 1.43] |  | 1.34 [0.86, 2.09] | 0.93 [0.65, 1.33] | 1.17 [0.86, 1.59] |
| Quinolinic acid | HMDB0000232 | 0.91 [0.51, 1.63] | 1.72 [1.22, 2.42] | 1.17 [0.81, 1.70] |  | 0.90 [0.53, 1.50] | 1.16 [0.80, 1.67] | 1.07 [0.75, 1.54] |  | 0.75 [0.47, 1.19] | 1.12 [0.83, 1.51] | 0.98 [0.72, 1.33] |  | 1.03 [0.62, 1.73] | 1.36 [0.97, 1.91] | 1.22 [0.90, 1.66] |
| **Phenylpropanoids and polyketides** | | | | | | | | | | | | | | | | |
| Warfarin | HMDB0001935 | 0.85 [0.40, 1.81] | 0.95 [0.48, 1.88] | 0.74 [0.33, 1.63] |  | 1.33 [0.82, 2.18] | 1.37 [0.93, 2.03] | 1.62 [1.14, 2.30] |  | 1.06 [0.80, 1.42] | 1.00 [0.78, 1.27] | 1.03 [0.82, 1.29] |  | 0.86 [0.51, 1.44] | 1.14 [0.86, 1.53] | 1.28 [1.01, 1.62] |
| Carboxyibuprofen | | 0.14 [0.03, 0.69] | 1.38 [1.02, 1.88] | 1.08 [0.74, 1.57] |  | 1.17 [0.88, 1.56] | 1.07 [0.82, 1.38] | 0.72 [0.46, 1.11] |  | 1.36 [1.02, 1.81] | 1.15 [0.90, 1.47] | 0.97 [0.73, 1.30] |  | 1.13 [0.75, 1.71] | 1.11 [0.84, 1.47] | 0.94 [0.70, 1.28] |

**eTable 4 Associations between metabolites and weight change groups across BMI categories older adults from the Health ABC study (N = 1536)** *Statistically significant interaction between metabolite and BMI after adjusting for age, race, and sex but not multiple comparisons (p<0.05). There was no significant interaction after adjusting for multiple comparisons (all FDRs>0.05).

| **Metabolites** | **HMDB ID** | **BMI<25 kg/m2** | | | **BMI25-30 kg/m2** | | | **BMI>=30 kg/m2** | | |
| --- | --- | --- | --- | --- | --- | --- | --- | --- | --- | --- |
|  |  | **Intentional weight loss** | **Unintentional weight loss** | **Weight gain** | **Intentional weight loss** | **Unintentional weight loss** | **Weight gain** | **Intentional weight loss** | **Unintentional weight loss** | **Weight gain** |
| **Lipids: Triradylcglycerols** | | | | | | | |  |  |  |
| TG(56:8) | HMDB0005392 | 1.77 [0.92, 3.41] | 0.73 [0.58, 0.91] | 0.84 [0.66, 1.07] | 0.95 [0.72, 1.25] | 0.74 [0.58, 0.96] | 0.79 [0.64, 0.98] | 0.90 [0.59, 1.37] | 0.77 [0.52, 1.16] | 1.23 [0.83, 1.82] |
| TG(56:7) * | HMDB0005462 | 1.53 [0.81, 2.88] | 0.74 [0.59, 0.92] | 0.74 [0.57, 0.94] | 0.91 [0.69, 1.20] | 0.79 [0.62, 1.01] | 0.84 [0.68, 1.04] | 1.00 [0.67, 1.49] | 0.82 [0.56, 1.21] | 1.25 [0.87, 1.79] |
| TG(58:10) | HMDB0005476 | 1.53 [0.79, 2.99] | 0.76 [0.61, 0.96] | 0.87 [0.68, 1.10] | 0.95 [0.71, 1.27] | 0.79 [0.61, 1.02] | 0.82 [0.66, 1.03] | 0.97 [0.63, 1.50] | 0.83 [0.55, 1.25] | 1.28 [0.86, 1.90] |
| TG(58:11) | HMDB0010531 | 1.49 [0.80, 2.78] | 0.77 [0.62, 0.96] | 0.84 [0.66, 1.07] | 0.97 [0.73, 1.29] | 0.81 [0.63, 1.05] | 0.81 [0.64, 1.01] | 1.01 [0.65, 1.56] | 0.82 [0.54, 1.25] | 1.22 [0.82, 1.82] |
| TG(60:12) | HMDB0005478 | 1.65 [0.86, 3.18] | 0.78 [0.63, 0.97] | 0.89 [0.70, 1.13] | 0.91 [0.69, 1.20] | 0.88 [0.69, 1.13] | 0.79 [0.63, 0.97] | 0.89 [0.56, 1.42] | 0.71 [0.45, 1.10] | 1.32 [0.86, 2.03] |
| TG(56:10) * | HMDB0010513 | 1.52 [0.79, 2.92] | 0.78 [0.63, 0.98] | 0.82 [0.64, 1.04] | 0.93 [0.69, 1.26] | 0.81 [0.62, 1.05] | 0.81 [0.64, 1.02] | 1.06 [0.70, 1.60] | 0.88 [0.59, 1.31] | 1.09 [0.74, 1.59] |
| TG(58:9) | HMDB0005463 | 1.46 [0.78, 2.70] | 0.78 [0.63, 0.98] | 0.89 [0.70, 1.13] | 0.92 [0.70, 1.22] | 0.85 [0.66, 1.09] | 0.83 [0.67, 1.02] | 0.89 [0.58, 1.38] | 0.76 [0.49, 1.16] | 1.36 [0.92, 2.01] |
| TG(54:9) | HMDB0010498 | 1.40 [0.67, 2.93] | 0.78 [0.63, 0.98] | 0.81 [0.63, 1.03] | 0.91 [0.67, 1.23] | 0.85 [0.65, 1.11] | 0.85 [0.67, 1.07] | 1.04 [0.69, 1.56] | 0.83 [0.57, 1.19] | 1.02 [0.71, 1.47] |
| TG(54:6) | HMDB0005391 | 1.09 [0.54, 2.19] | 0.87 [0.70, 1.09] | 1.01 [0.79, 1.28] | 0.93 [0.69, 1.25] | 0.66 [0.52, 0.85] | 1.01 [0.80, 1.27] | 1.12 [0.74, 1.68] | 0.98 [0.67, 1.44] | 0.87 [0.62, 1.23] |
| TG(53:3) | HMDB0043058 | 1.59 [0.70, 3.61] | 0.94 [0.75, 1.19] | 0.79 [0.62, 1.02] | 0.85 [0.64, 1.12] | 0.75 [0.59, 0.95] | 0.95 [0.76, 1.19] | 1.72 [1.07, 2.76] | 0.73 [0.48, 1.10] | 0.93 [0.63, 1.35] |
| TG(54:5) | HMDB0005385 | 1.11 [0.54, 2.27] | 0.88 [0.70, 1.11] | 0.92 [0.72, 1.17] | 1.00 [0.74, 1.35] | 0.70 [0.54, 0.90] | 1.04 [0.82, 1.31] | 1.07 [0.70, 1.65] | 0.92 [0.62, 1.36] | 0.89 [0.62, 1.27] |
| TG(52:7) | HMDB0010517 | 1.28 [0.61, 2.66] | 0.80 [0.63, 1.01] | 0.77 [0.60, 1.00] | 0.92 [0.68, 1.25] | 0.91 [0.70, 1.18] | 0.89 [0.71, 1.12] | 1.13 [0.76, 1.70] | 0.94 [0.65, 1.37] | 0.89 [0.63, 1.25] |
| TG(54:2) | HMDB0005403 | 1.53 [0.77, 3.04] | 1.02 [0.82, 1.26] | 0.88 [0.69, 1.12] | 1.25 [0.93, 1.68] | 1.13 [0.88, 1.45] | 1.08 [0.86, 1.34] | 1.24 [0.82, 1.86] | 0.85 [0.59, 1.24] | 0.76 [0.54, 1.06] |
| TG(44:0) | HMDB0042063 | 0.69 [0.34, 1.39] | 1.07 [0.85, 1.34] | 0.83 [0.65, 1.06] | 1.21 [0.90, 1.64] | 1.30 [1.00, 1.70] | 1.10 [0.88, 1.39] | 1.13 [0.76, 1.69] | 1.14 [0.79, 1.66] | 0.90 [0.64, 1.26] |
| TG(46:0) * | HMDB0010411 | 0.78 [0.42, 1.44] | 1.05 [0.84, 1.31] | 0.88 [0.70, 1.11] | 1.21 [0.89, 1.64] | 1.39 [1.05, 1.83] | 1.06 [0.84, 1.32] | 1.11 [0.73, 1.69] | 1.16 [0.78, 1.74] | 0.91 [0.65, 1.26] |
| TG(46:1) | HMDB0010412 | 0.71 [0.35, 1.42] | 1.07 [0.85, 1.35] | 0.82 [0.64, 1.05] | 1.17 [0.86, 1.59] | 1.38 [1.05, 1.80] | 1.07 [0.84, 1.35] | 1.10 [0.73, 1.64] | 1.12 [0.77, 1.65] | 0.87 [0.62, 1.23] |
| **Lipids: Glycerophosphocholines** | | | | | | | |  |  |  |
| PC(38:6) | HMDB0007991 | 3.29 [1.41, 7.66] | 0.69 [0.55, 0.88] | 0.73 [0.57, 0.94] | 1.06 [0.80, 1.41] | 0.89 [0.70, 1.14] | 0.91 [0.73, 1.13] | 0.82 [0.52, 1.28] | 0.80 [0.53, 1.21] | 0.86 [0.59, 1.24] |
| PC(40:6) | HMDB0008057 | 3.31 [1.52, 7.19] | 0.72 [0.56, 0.92] | 0.72 [0.56, 0.95] | 1.04 [0.78, 1.38] | 0.91 [0.71, 1.17] | 0.84 [0.67, 1.05] | 0.85 [0.55, 1.30] | 0.76 [0.51, 1.12] | 0.89 [0.62, 1.29] |
| PC(40:9) | HMDB0008731 | 3.88 [1.47, 10.23] | 0.72 [0.58, 0.91] | 0.75 [0.58, 0.96] | 1.05 [0.79, 1.40] | 0.89 [0.70, 1.14] | 0.89 [0.71, 1.10] | 0.86 [0.55, 1.33] | 0.79 [0.53, 1.19] | 0.88 [0.61, 1.29] |
| PC(P-38:6)/PC(O-38:7) | HMDB0011229 | 1.33 [0.63, 2.80] | 0.72 [0.56, 0.92] | 0.83 [0.64, 1.08] | 0.98 [0.74, 1.31] | 0.88 [0.68, 1.14] | 0.93 [0.75, 1.17] | 0.71 [0.48, 1.07] | 1.17 [0.77, 1.77] | 0.90 [0.62, 1.29] |
| PC(36:0) | HMDB0008036 | 1.79 [0.91, 3.51] | 0.94 [0.74, 1.19] | 0.95 [0.73, 1.23] | 0.98 [0.73, 1.32] | 0.70 [0.55, 0.89] | 0.83 [0.66, 1.04] | 0.88 [0.61, 1.27] | 1.04 [0.74, 1.47] | 0.88 [0.65, 1.20] |
| LPC(24:0) | HMDB0010405 | 1.44 [0.71, 2.94] | 0.83 [0.65, 1.06] | 0.90 [0.69, 1.17] | 0.96 [0.72, 1.28] | 0.72 [0.57, 0.92] | 0.86 [0.69, 1.07] | 1.14 [0.79, 1.64] | 1.13 [0.80, 1.60] | 0.96 [0.69, 1.32] |
| PC(36:2) | HMDB0008039 | 1.25 [0.55, 2.83] | 0.82 [0.63, 1.06] | 0.89 [0.67, 1.18] | 1.04 [0.78, 1.39] | 0.85 [0.68, 1.06] | 0.84 [0.69, 1.03] | 0.91 [0.61, 1.34] | 1.18 [0.81, 1.73] | 0.73 [0.53, 1.00] |
| PC(36:4)_A | HMDB0007983 | 0.82 [0.39, 1.74] | 0.83 [0.64, 1.06] | 0.89 [0.68, 1.17] | 0.89 [0.67, 1.19] | 0.78 [0.61, 1.00] | 0.84 [0.68, 1.05] | 1.05 [0.69, 1.59] | 1.26 [0.85, 1.87] | 0.72 [0.51, 1.00] |
| PC(32:2) | HMDB0007874 | 0.58 [0.26, 1.29] | 0.80 [0.61, 1.05] | 0.74 [0.55, 0.99] | 1.15 [0.80, 1.64] | 1.11 [0.82, 1.50] | 0.88 [0.67, 1.14] | 0.94 [0.61, 1.46] | 1.23 [0.81, 1.85] | 0.79 [0.55, 1.14] |
| LPC(14:0) | HMDB0010379 | 0.61 [0.28, 1.31] | 0.96 [0.74, 1.25] | 0.71 [0.54, 0.94] | 1.15 [0.84, 1.57] | 1.06 [0.81, 1.38] | 0.96 [0.76, 1.21] | 1.11 [0.74, 1.66] | 1.02 [0.70, 1.48] | 0.75 [0.53, 1.06] |
| PC(36:1) | HMDB0008038 | 1.31 [0.60, 2.85] | 0.92 [0.71, 1.19] | 0.75 [0.57, 1.00] | 0.99 [0.74, 1.32] | 1.05 [0.82, 1.35] | 0.86 [0.69, 1.07] | 1.18 [0.80, 1.74] | 1.14 [0.81, 1.60] | 0.87 [0.64, 1.19] |
| PC(30:1) | HMDB0007870 | 0.60 [0.29, 1.27] | 0.97 [0.75, 1.25] | 0.76 [0.57, 0.99] | 1.12 [0.80, 1.57] | 1.53 [1.14, 2.05] | 0.96 [0.74, 1.25] | 0.97 [0.64, 1.47] | 1.16 [0.79, 1.72] | 0.83 [0.58, 1.18] |
| LPC(22:5) | HMDB0010402 | 0.57 [0.38, 0.87] | 1.13 [0.89, 1.42] | 1.01 [0.79, 1.29] | 0.97 [0.73, 1.28] | 1.35 [1.02, 1.78] | 1.21 [0.96, 1.53] | 1.27 [0.79, 2.04] | 1.56 [0.98, 2.48] | 0.77 [0.54, 1.09] |
| **Lipids: Phosphosphingolipids** | | | | | | | |  |  |  |
| SM(d18:1/24:0) | HMDB0011697 | 1.62 [0.76, 3.43] | 0.85 [0.67, 1.09] | 0.94 [0.72, 1.23] | 1.03 [0.77, 1.37] | 0.71 [0.57, 0.90] | 0.80 [0.65, 0.98] | 0.90 [0.61, 1.34] | 0.96 [0.67, 1.39] | 0.97 [0.70, 1.36] |
| SM(d18:1/22:0) | HMDB0012103 | 1.72 [0.79, 3.73] | 0.88 [0.69, 1.13] | 1.00 [0.76, 1.30] | 1.01 [0.76, 1.35] | 0.73 [0.58, 0.92] | 0.83 [0.67, 1.03] | 0.91 [0.61, 1.36] | 1.01 [0.69, 1.48] | 0.93 [0.66, 1.30] |
| SM(d18:1/20:0) | HMDB0012102 | 1.59 [0.72, 3.53] | 0.92 [0.72, 1.18] | 0.97 [0.74, 1.28] | 1.00 [0.75, 1.34] | 0.74 [0.59, 0.94] | 0.87 [0.71, 1.08] | 0.93 [0.62, 1.40] | 0.96 [0.66, 1.40] | 0.98 [0.70, 1.38] |
| SM(d18:1/16:1) | HMDB0240613 | 2.16 [0.90, 5.17] | 0.87 [0.66, 1.14] | 1.05 [0.78, 1.42] | 0.90 [0.68, 1.20] | 0.80 [0.63, 1.01] | 0.84 [0.67, 1.05] | 0.85 [0.55, 1.30] | 0.97 [0.65, 1.43] | 0.87 [0.61, 1.24] |
| SM(d18:1/22:1) | HMDB0012104 | 2.20 [0.93, 5.20] | 0.88 [0.67, 1.14] | 1.04 [0.78, 1.39] | 0.95 [0.71, 1.28] | 0.76 [0.60, 0.97] | 0.85 [0.69, 1.06] | 0.89 [0.60, 1.33] | 1.04 [0.71, 1.52] | 1.02 [0.73, 1.43] |
| **Lipids: Glycerophosphoethanolamines** | | | | | | | |  |  |  |
| PE(P-38:6)/PE(O-38:7) | HMDB0011420 | 1.70 [0.80, 3.62] | 0.77 [0.60, 0.98] | 0.81 [0.62, 1.06] | 1.13 [0.84, 1.52] | 0.87 [0.67, 1.14] | 0.87 [0.69, 1.10] | 0.82 [0.51, 1.31] | 0.82 [0.53, 1.28] | 0.89 [0.59, 1.32] |
| PE(38:2) | HMDB0008942 | 1.65 [0.76, 3.59] | 0.89 [0.69, 1.14] | 0.86 [0.66, 1.13] | 0.99 [0.74, 1.32] | 0.78 [0.62, 0.98] | 0.90 [0.73, 1.12] | 1.23 [0.82, 1.83] | 0.99 [0.69, 1.42] | 0.84 [0.60, 1.16] |
| LPE(20:4) | HMDB0011517 | 0.85 [0.42, 1.73] | 1.18 [0.93, 1.50] | 0.97 [0.75, 1.25] | 1.16 [0.87, 1.55] | 1.20 [0.94, 1.54] | 1.09 [0.88, 1.35] | 1.25 [0.85, 1.83] | 1.24 [0.88, 1.75] | 0.87 [0.64, 1.20] |
| **Lipids: Glycerophosphoserines** | | | | | | | |  |  |  |
| PS(34:0) * | HMDB0012356 | 1.67 [0.84, 3.33] | 0.76 [0.60, 0.96] | 0.81 [0.62, 1.05] | 0.97 [0.74, 1.29] | 0.91 [0.71, 1.15] | 0.82 [0.67, 1.02] | 0.96 [0.63, 1.48] | 0.96 [0.64, 1.44] | 0.96 [0.66, 1.39] |
| **Lipids: Fatty acids and conjugates** | | | | | | | |  |  |  |
| Azelaic acid | HMDB0000784 | 0.99 [0.49, 2.04] | 1.07 [0.84, 1.36] | 0.89 [0.70, 1.12] | 0.88 [0.67, 1.16] | 1.14 [0.89, 1.48] | 0.84 [0.68, 1.03] | 0.89 [0.65, 1.22] | 1.01 [0.72, 1.42] | 0.82 [0.64, 1.06] |
| Adipic acid or Methylglutaric acid | HMDB0000448 | 0.85 [0.34, 2.08] | 0.97 [0.73, 1.28] | 1.39 [1.04, 1.86] | 0.96 [0.71, 1.29] | 1.21 [0.95, 1.53] | 1.12 [0.90, 1.40] | 1.38 [0.88, 2.15] | 1.45 [0.97, 2.16] | 1.09 [0.75, 1.59] |
| 3-Methyladipic acid or Pimelic acid | HMDB0000555 | 0.77 [0.32, 1.85] | 1.19 [0.94, 1.52] | 1.06 [0.80, 1.39] | 0.96 [0.71, 1.31] | 1.20 [0.93, 1.54] | 0.89 [0.70, 1.13] | 1.12 [0.74, 1.69] | 1.47 [1.02, 2.13] | 0.80 [0.57, 1.14] |
| Suberic acid | HMDB0000893 | 1.00 [0.44, 2.31] | 1.16 [0.89, 1.50] | 1.33 [1.00, 1.76] | 0.68 [0.48, 0.96] | 1.23 [0.96, 1.56] | 0.97 [0.76, 1.23] | 0.90 [0.58, 1.39] | 1.18 [0.82, 1.69] | 1.07 [0.75, 1.51] |
| Juniperic acid | HMDB0006294 | 1.01 [0.55, 1.83] | 1.12 [0.90, 1.39] | 1.15 [0.90, 1.47] | 1.08 [0.81, 1.45] | 1.37 [1.07, 1.77] | 1.20 [0.96, 1.50] | 0.76 [0.49, 1.19] | 1.14 [0.78, 1.67] | 1.09 [0.76, 1.55] |
| Lauric acid | HMDB0000638 | 0.78 [0.40, 1.51] | 1.08 [0.87, 1.35] | 1.08 [0.85, 1.38] | 1.13 [0.83, 1.55] | 1.45 [1.11, 1.89] | 1.19 [0.93, 1.51] | 0.78 [0.50, 1.21] | 1.17 [0.80, 1.71] | 1.16 [0.82, 1.65] |
| Tetradecanedioic acid* | HMDB0000872 | 0.70 [0.30, 1.67] | 0.97 [0.77, 1.23] | 1.11 [0.87, 1.40] | 1.18 [0.90, 1.56] | 1.44 [1.15, 1.79] | 1.23 [0.99, 1.52] | 0.69 [0.41, 1.18] | 1.34 [0.96, 1.87] | 1.02 [0.71, 1.46] |
| Hexadecanedioic acid* | HMDB0000672 | 0.93 [0.44, 1.99] | 1.00 [0.78, 1.27] | 1.26 [0.98, 1.63] | 1.14 [0.87, 1.48] | 1.42 [1.15, 1.75] | 1.22 [0.99, 1.49] | 0.85 [0.55, 1.31] | 1.25 [0.89, 1.76] | 0.95 [0.67, 1.36] |
| Sebacic acid | HMDB0000792 | 0.89 [0.43, 1.85] | 1.13 [0.89, 1.43] | 1.08 [0.84, 1.40] | 0.82 [0.62, 1.10] | 1.43 [1.12, 1.81] | 0.95 [0.77, 1.19] | 1.09 [0.75, 1.59] | 1.32 [0.92, 1.90] | 1.13 [0.81, 1.57] |
| **Lipids: Steroid esters** | | | | | | | | | | |
| CE(20:5) | HMDB0006731 | 2.71 [1.27, 5.77] | 0.83 [0.67, 1.04] | 0.90 [0.71, 1.16] | 0.91 [0.69, 1.21] | 0.92 [0.72, 1.18] | 0.83 [0.67, 1.03] | 0.76 [0.51, 1.15] | 0.73 [0.49, 1.09] | 1.14 [0.78, 1.66] |
| CE(18:0) | HMDB0010368 | 1.38 [0.64, 2.97] | 0.96 [0.76, 1.23] | 1.00 [0.77, 1.30] | 0.77 [0.59, 1.00] | 0.73 [0.58, 0.92] | 0.89 [0.72, 1.11] | 0.78 [0.53, 1.13] | 0.97 [0.68, 1.38] | 0.85 [0.62, 1.17] |
| CE(22:6) | HMDB0006733 | 3.31 [1.19, 9.22] | 0.86 [0.68, 1.08] | 1.02 [0.78, 1.33] | 0.93 [0.68, 1.26] | 0.88 [0.67, 1.16] | 0.82 [0.65, 1.04] | 0.69 [0.46, 1.03] | 0.76 [0.52, 1.11] | 1.08 [0.74, 1.56] |
| CE(22:4) | HMDB0006729 | 0.91 [0.41, 2.02] | 0.92 [0.70, 1.21] | 1.07 [0.78, 1.46] | 0.78 [0.62, 0.97] | 0.87 [0.69, 1.10] | 1.06 [0.83, 1.35] | 0.86 [0.61, 1.21] | 1.13 [0.76, 1.67] | 1.10 [0.77, 1.58] |
| **Lipids: Fatty acid esters** | | | | | | | |  |  |  |
| CAR(14:2) * | HMDB0013331 | 0.57 [0.29, 1.12] | 0.83 [0.66, 1.04] | 1.05 [0.82, 1.34] | 0.89 [0.67, 1.18] | 1.10 [0.85, 1.41] | 1.11 [0.89, 1.39] | 0.72 [0.49, 1.04] | 1.25 [0.88, 1.79] | 0.98 [0.71, 1.36] |
| CAR(6:0) | HMDB0000705 | 0.75 [0.35, 1.59] | 1.15 [0.91, 1.45] | 1.02 [0.79, 1.31] | 1.05 [0.78, 1.41] | 1.16 [0.90, 1.49] | 1.11 [0.89, 1.39] | 0.84 [0.56, 1.26] | 1.25 [0.92, 1.70] | 1.13 [0.84, 1.53] |
| CAR(4:0(OH)) | HMDB0013127 | 0.67 [0.37, 1.20] | 1.04 [0.84, 1.28] | 1.01 [0.80, 1.26] | 1.20 [0.88, 1.63] | 1.37 [1.05, 1.78] | 1.29 [1.02, 1.64] | 0.78 [0.52, 1.16] | 1.45 [0.99, 2.12] | 1.24 [0.88, 1.76] |
| **Lipids: Bile Acids** | | | | | | | | | | |
| Hyodeoxycholic acid/Ursodeoxycholic acid | HMDB0000733 | 0.96 [0.48, 1.93] | 1.08 [0.85, 1.36] | 1.17 [0.91, 1.52] | 1.41 [1.05, 1.88] | 1.06 [0.83, 1.35] | 1.14 [0.92, 1.42] | 1.36 [0.91, 2.03] | 1.00 [0.68, 1.47] | 0.89 [0.63, 1.26] |
| **Organic acids: Amino acids, peptides, and analogues** | | | | | | | | | | |
| Tryptophan | HMDB0000929 | 0.57 [0.30, 1.09] | 0.76 [0.59, 0.97] | 1.07 [0.81, 1.42] | 1.16 [0.82, 1.62] | 0.94 [0.70, 1.25] | 0.99 [0.76, 1.28] | 0.65 [0.46, 0.91] | 0.74 [0.53, 1.04] | 0.84 [0.61, 1.17] |
| Valine | HMDB0000883 | 0.66 [0.36, 1.19] | 0.79 [0.62, 1.00] | 0.88 [0.68, 1.15] | 1.14 [0.84, 1.56] | 0.93 [0.72, 1.21] | 0.88 [0.70, 1.11] | 0.81 [0.58, 1.14] | 0.89 [0.63, 1.27] | 0.76 [0.56, 1.02] |
| Homoarginine | HMDB0000670 | 0.91 [0.41, 2.01] | 0.71 [0.55, 0.92] | 0.81 [0.62, 1.07] | 0.96 [0.72, 1.28] | 0.94 [0.73, 1.20] | 0.88 [0.71, 1.09] | 0.76 [0.54, 1.07] | 0.98 [0.68, 1.41] | 0.93 [0.67, 1.30] |
| Asparagine | HMDB0000168 | 0.62 [0.35, 1.10] | 0.75 [0.59, 0.96] | 0.82 [0.63, 1.07] | 1.02 [0.77, 1.36] | 0.98 [0.76, 1.26] | 0.82 [0.66, 1.01] | 0.85 [0.64, 1.11] | 0.98 [0.70, 1.38] | 0.99 [0.72, 1.36] |
| Dimethylglycine | HMDB0000092 | 0.62 [0.28, 1.36] | 1.06 [0.83, 1.37] | 1.20 [0.91, 1.56] | 1.10 [0.82, 1.48] | 1.22 [0.95, 1.56] | 1.10 [0.88, 1.38] | 1.17 [0.84, 1.64] | 1.21 [0.88, 1.65] | 1.22 [0.91, 1.63] |
| N-Formylmethionine | HMDB0001015 | 0.92 [0.48, 1.77] | 1.62 [1.20, 2.19] | 1.21 [0.89, 1.66] | 1.32 [0.95, 1.84] | 1.08 [0.81, 1.42] | 1.08 [0.83, 1.39] | 1.06 [0.69, 1.64] | 1.10 [0.73, 1.64] | 0.85 [0.58, 1.24] |
| Ornithine | HMDB0000214 | 1.21 [0.58, 2.52] | 1.28 [1.00, 1.62] | 1.07 [0.83, 1.37] | 1.02 [0.76, 1.35] | 1.10 [0.86, 1.42] | 0.82 [0.67, 1.01] | 0.85 [0.64, 1.14] | 1.23 [0.84, 1.80] | 1.01 [0.74, 1.39] |
| N-Acetylglutamic acid | HMDB0001138 | 0.44 [0.18, 1.06] | 1.40 [1.09, 1.80] | 1.21 [0.92, 1.60] | 1.00 [0.73, 1.38] | 1.23 [0.94, 1.60] | 0.80 [0.62, 1.03] | 1.36 [0.91, 2.03] | 1.11 [0.76, 1.62] | 0.99 [0.71, 1.40] |
| **Organic acids: Other** | | | | | | | |  |  |  |
| Levulinic acid | HMDB0000720 | 1.00 [0.50, 2.01] | 0.94 [0.78, 1.14] | 0.95 [0.77, 1.18] | 1.36 [0.97, 1.92] | 0.98 [0.73, 1.32] | 0.80 [0.61, 1.04] | 0.72 [0.46, 1.12] | 0.70 [0.47, 1.06] | 0.70 [0.48, 1.02] |
| N-Acetylcarnosine | HMDB0012881 | 1.25 [0.50, 3.12] | 0.95 [0.72, 1.26] | 0.91 [0.67, 1.22] | 0.97 [0.68, 1.40] | 0.97 [0.71, 1.33] | 0.76 [0.59, 0.97] | 1.08 [0.64, 1.80] | 0.91 [0.60, 1.39] | 0.78 [0.56, 1.10] |
| Ketoisovaleric acid* | HMDB0000019 | 0.55 [0.26, 1.16] | 0.87 [0.69, 1.10] | 0.83 [0.64, 1.08] | 0.91 [0.68, 1.23] | 1.11 [0.86, 1.43] | 0.80 [0.64, 1.00] | 0.91 [0.62, 1.34] | 1.16 [0.81, 1.66] | 0.92 [0.66, 1.29] |
| 2-Hydroxyglutaric acid | HMDB0000694 | 0.73 [0.31, 1.76] | 1.16 [0.89, 1.50] | 1.05 [0.79, 1.40] | 0.91 [0.68, 1.23] | 1.17 [0.92, 1.49] | 0.98 [0.79, 1.23] | 1.16 [0.77, 1.74] | 1.30 [0.90, 1.89] | 0.87 [0.62, 1.20] |
| cis-Aconitic acid | HMDB0000072 | 0.72 [0.33, 1.57] | 1.01 [0.78, 1.30] | 1.27 [0.96, 1.67] | 1.02 [0.74, 1.39] | 1.43 [1.09, 1.86] | 1.15 [0.91, 1.46] | 0.82 [0.55, 1.22] | 1.18 [0.82, 1.71] | 0.87 [0.63, 1.20] |
| 3S-Hydroxyhexanoic acid* | HMDB0010718 | 1.08 [0.51, 2.30] | 0.99 [0.77, 1.26] | 1.18 [0.90, 1.53] | 1.15 [0.84, 1.57] | 1.40 [1.07, 1.83] | 1.21 [0.95, 1.54] | 0.76 [0.53, 1.07] | 1.55 [1.05, 2.28] | 1.08 [0.77, 1.50] |
| 3-Hydroxyoctanoic acid* | HMDB0001954 | 0.92 [0.43, 2.00] | 1.03 [0.81, 1.32] | 1.18 [0.90, 1.55] | 1.09 [0.81, 1.47] | 1.42 [1.09, 1.85] | 1.23 [0.98, 1.56] | 0.81 [0.58, 1.13] | 1.28 [0.89, 1.86] | 0.89 [0.66, 1.20] |
| N1-Acetylspermidine | HMDB0001276 | 0.89 [0.43, 1.86] | 1.11 [0.87, 1.41] | 1.31 [1.01, 1.69] | 1.12 [0.81, 1.54] | 1.36 [1.03, 1.79] | 1.25 [0.98, 1.60] | 0.89 [0.64, 1.24] | 1.41 [0.99, 2.00] | 1.23 [0.89, 1.71] |
| Fumaric acid or Maleic acid | HMDB0000134 | 0.82 [0.37, 1.85] | 1.10 [0.87, 1.39] | 1.16 [0.90, 1.49] | 1.08 [0.80, 1.44] | 1.38 [1.08, 1.76] | 1.10 [0.88, 1.37] | 0.93 [0.62, 1.40] | 1.28 [0.88, 1.86] | 0.81 [0.57, 1.15] |
| Malic acid | HMDB0000156 | 0.75 [0.33, 1.72] | 1.13 [0.89, 1.43] | 1.15 [0.88, 1.49] | 1.11 [0.83, 1.48] | 1.39 [1.09, 1.78] | 1.10 [0.88, 1.37] | 0.85 [0.57, 1.28] | 1.31 [0.90, 1.90] | 0.87 [0.62, 1.23] |
| **Nucleosides, nucleotides, and analogues** | | | | | | | |  |  |  |
| Uridine | HMDB0000296 | 0.77 [0.42, 1.43] | 0.77 [0.63, 0.96] | 0.92 [0.72, 1.16] | 0.93 [0.69, 1.25] | 0.71 [0.56, 0.90] | 0.89 [0.71, 1.11] | 0.81 [0.57, 1.16] | 0.74 [0.52, 1.05] | 0.86 [0.62, 1.20] |
| ADP | HMDB0001341 | 2.14 [0.66, 6.97] | 0.79 [0.64, 0.99] | 0.89 [0.69, 1.15] | 1.19 [0.84, 1.70] | 0.91 [0.70, 1.18] | 1.02 [0.80, 1.30] | 0.80 [0.58, 1.10] | 0.84 [0.63, 1.12] | 0.97 [0.72, 1.31] |
| ATP | HMDB0000538 | 2.97 [0.63, 14.06] | 0.84 [0.67, 1.05] | 0.93 [0.72, 1.21] | 1.13 [0.79, 1.61] | 0.91 [0.71, 1.17] | 1.05 [0.82, 1.35] | 0.78 [0.59, 1.03] | 0.80 [0.61, 1.05] | 0.98 [0.72, 1.33] |
| N4-Acetylcytidine | HMDB0005923 | 0.59 [0.27, 1.31] | 1.08 [0.85, 1.37] | 1.08 [0.83, 1.40] | 0.96 [0.70, 1.32] | 1.34 [1.03, 1.75] | 1.14 [0.90, 1.45] | 0.92 [0.62, 1.36] | 1.27 [0.89, 1.81] | 1.13 [0.81, 1.57] |
| Pseudouridine | HMDB0000767 | 0.64 [0.26, 1.53] | 1.22 [0.93, 1.60] | 1.23 [0.91, 1.65] | 0.92 [0.65, 1.30] | 1.44 [1.09, 1.90] | 0.93 [0.72, 1.21] | 1.19 [0.79, 1.79] | 1.17 [0.79, 1.71] | 1.19 [0.85, 1.68] |
| **Organic oxygen compounds** | | | | | | | |  |  |  |
| Myo-inositol | HMDB0000211 | 0.54 [0.19, 1.58] | 1.30 [1.04, 1.61] | 1.01 [0.76, 1.33] | 0.96 [0.70, 1.31] | 1.07 [0.84, 1.38] | 1.21 [0.98, 1.49] | 1.04 [0.70, 1.55] | 0.97 [0.66, 1.43] | 0.75 [0.50, 1.12] |
| Glyceric acid | HMDB0000139 | 0.87 [0.42, 1.79] | 0.83 [0.66, 1.05] | 1.05 [0.83, 1.33] | 0.95 [0.71, 1.27] | 0.79 [0.61, 1.03] | 0.81 [0.64, 1.02] | 1.55 [1.10, 2.20] | 0.90 [0.60, 1.34] | 1.00 [0.70, 1.42] |
| Hexose | HMDB0000122 | 0.74 [0.32, 1.75] | 1.36 [1.07, 1.74] | 1.10 [0.83, 1.46] | 0.80 [0.58, 1.11] | 1.11 [0.86, 1.43] | 1.14 [0.91, 1.43] | 1.07 [0.78, 1.47] | 0.94 [0.69, 1.29] | 0.79 [0.58, 1.08] |
| Adonitol or Arabitol | HMDB0000508 | 0.65 [0.26, 1.66] | 1.27 [0.99, 1.63] | 1.07 [0.81, 1.43] | 0.93 [0.66, 1.31] | 1.27 [0.96, 1.69] | 0.91 [0.69, 1.18] | 1.13 [0.76, 1.68] | 1.03 [0.71, 1.48] | 1.08 [0.78, 1.50] |
| Glucuronic acid | HMDB0000127 | 0.93 [0.40, 2.15] | 1.11 [0.88, 1.41] | 0.92 [0.69, 1.22] | 1.14 [0.85, 1.54] | 1.22 [0.95, 1.57] | 1.06 [0.83, 1.35] | 1.44 [1.05, 1.98] | 1.22 [0.90, 1.66] | 1.00 [0.74, 1.36] |
| Sorbitol | HMDB0000247 | 0.65 [0.23, 1.87] | 1.13 [0.90, 1.43] | 1.15 [0.89, 1.47] | 1.13 [0.87, 1.48] | 1.38 [1.11, 1.70] | 1.00 [0.80, 1.26] | 0.87 [0.57, 1.33] | 1.06 [0.76, 1.47] | 1.09 [0.82, 1.46] |
| Threitol | HMDB0004136 | 0.55 [0.22, 1.33] | 1.36 [1.05, 1.76] | 1.22 [0.92, 1.63] | 1.09 [0.80, 1.49] | 1.32 [1.02, 1.71] | 1.06 [0.83, 1.36] | 0.99 [0.67, 1.49] | 1.18 [0.82, 1.69] | 0.98 [0.71, 1.37] |
| Sucrose or Lactose or Trehalose | HMDB0000258 | 0.99 [0.46, 2.15] | 1.17 [0.93, 1.47] | 1.26 [0.99, 1.61] | 1.08 [0.79, 1.47] | 1.43 [1.12, 1.83] | 1.04 [0.81, 1.32] | 1.18 [0.82, 1.68] | 1.21 [0.86, 1.68] | 0.89 [0.64, 1.23] |
| **Organoheterocyclic compounds** | | | | | | | | | | |
| Uracil | HMDB0000300 | 0.70 [0.38, 1.29] | 0.78 [0.63, 0.96] | 0.87 [0.69, 1.10] | 0.89 [0.66, 1.20] | 0.70 [0.55, 0.90] | 0.88 [0.70, 1.10] | 0.81 [0.56, 1.17] | 0.78 [0.54, 1.12] | 0.84 [0.60, 1.17] |
| Niacinamide | HMDB0001406 | 1.22 [0.62, 2.42] | 0.71 [0.56, 0.90] | 0.71 [0.55, 0.92] | 1.06 [0.80, 1.40] | 0.81 [0.63, 1.03] | 1.22 [0.98, 1.52] | 0.86 [0.59, 1.25] | 0.95 [0.66, 1.36] | 0.91 [0.66, 1.26] |
| Serotonin | HMDB0000259 | 1.43 [0.45, 4.50] | 0.70 [0.55, 0.89] | 0.86 [0.64, 1.14] | 0.98 [0.73, 1.33] | 0.92 [0.71, 1.19] | 0.90 [0.73, 1.12] | 0.77 [0.57, 1.05] | 1.01 [0.71, 1.44] | 0.85 [0.64, 1.12] |
| 1-Methyl nicotinamide | HMDB0000699 | 0.74 [0.34, 1.63] | 0.81 [0.64, 1.03] | 0.88 [0.68, 1.12] | 0.97 [0.73, 1.30] | 0.85 [0.66, 1.11] | 1.00 [0.80, 1.25] | 1.04 [0.70, 1.52] | 0.91 [0.62, 1.33] | 0.91 [0.64, 1.30] |
| Acisoga | HMDB0061384 | 0.40 [0.21, 0.75] | 1.12 [0.88, 1.43] | 1.17 [0.90, 1.52] | 1.17 [0.86, 1.58] | 0.99 [0.76, 1.29] | 1.14 [0.90, 1.45] | 1.17 [0.80, 1.71] | 1.28 [0.89, 1.85] | 1.44 [1.01, 2.04] |
| Theophylline | HMDB0001889 | 0.81 [0.37, 1.76] | 1.27 [1.02, 1.58] | 1.02 [0.79, 1.31] | 1.10 [0.82, 1.46] | 0.99 [0.77, 1.28] | 1.21 [0.97, 1.51] | 1.01 [0.69, 1.49] | 1.23 [0.87, 1.75] | 0.84 [0.60, 1.18] |
| Glucosan or 3-Hydroxymethylglutaric acid | HMDB0000640 | 1.06 [0.45, 2.48] | 1.08 [0.83, 1.41] | 1.22 [0.93, 1.61] | 1.12 [0.81, 1.56] | 1.37 [1.05, 1.80] | 0.99 [0.75, 1.29] | 1.06 [0.68, 1.66] | 1.19 [0.79, 1.79] | 1.13 [0.78, 1.64] |
| Quinolinic acid | HMDB0000232 | 0.48 [0.18, 1.28] | 1.28 [0.98, 1.67] | 1.18 [0.88, 1.58] | 1.19 [0.87, 1.65] | 1.39 [1.08, 1.80] | 1.04 [0.80, 1.35] | 0.63 [0.42, 0.97] | 1.27 [0.88, 1.82] | 1.14 [0.82, 1.60] |
| **Benzenoids** | | | | | | | | | | |
| Homovanillic acid | HMDB0000118 | 0.58 [0.25, 1.32] | 1.13 [0.86, 1.47] | 1.24 [0.93, 1.65] | 0.86 [0.63, 1.18] | 1.33 [1.02, 1.72] | 0.99 [0.78, 1.25] | 0.95 [0.62, 1.45] | 1.23 [0.83, 1.83] | 0.99 [0.69, 1.42] |
| **Phenylpropanoids and polyketides** | | | | | | | |  |  |  |
| Warfarin | HMDB0001935 | 0.48 [0.07, 3.56] | 1.21 [0.94, 1.57] | 1.27 [0.97, 1.65] | 1.03 [0.78, 1.35] | 1.11 [0.89, 1.39] | 1.22 [1.02, 1.46] | 1.03 [0.74, 1.44] | 0.74 [0.42, 1.32] | 0.96 [0.67, 1.38] |
| Carboxyibuprofen |  | 1.13 [0.56, 2.28] | 1.23 [0.99, 1.53] | 0.84 [0.60, 1.19] | 1.09 [0.82, 1.44] | 1.11 [0.88, 1.40] | 0.96 [0.75, 1.23] | 1.23 [0.96, 1.57] | 1.14 [0.90, 1.46] | 0.94 [0.71, 1.26] |
